# Effects of genetically proxied lipid-lowering drugs on acute myocardial infarction: a drug-target mendelian randomization study

**DOI:** 10.1186/s12944-024-02133-w

**Published:** 2024-06-03

**Authors:** Wendi Xiao, Yueying Li, Zhenhuang Zhuang, Zimin Song, Wenxiu Wang, Ninghao Huang, Xue Dong, Jinzhu Jia, Zhonghua Liu, Yimin Zhao, Lu Qi, Tao Huang

**Affiliations:** 1 Department of Epidemiology and Biostatistics, School of Public Health, Peking University, 38 Xueyuan Road, Beijing, 100191 China; 2 Department of Biostatistics, School of Public Health, Peking University, Beijing, China; 3 Department of Statistics and Actuarial Science, The University of Hong Kong, Hong Kong, China; 4 Department of Epidemiology, School of Public Health and Tropical Medicine, Tulane University, New Orleans, LA USA; 5 Department of Nutrition, Harvard T.H. Chan School of Public Health, Boston, MA USA; 6 Key Laboratory of Molecular Cardiovascular Sciences (Peking University), Ministry of Education, Beijing, China; 7 Center for Intelligent Public Health, Academy for Artificial Intelligence, Peking University, Beijing, China

**Keywords:** Mendelian randomization, Lipid-lowering agents, Coronary artery disease

## Abstract

**Objective:**

High low-density-lipoprotein (LDL) cholesterol has been associated with an increased risk of coronary artery diseases (CAD) including acute myocardial infarction (AMI). However, whether lipids lowering drug treatment is causally associated with decreased risk of AMI remains largely unknown. We used Mendelian randomization (MR) to evaluate the influence of genetic variation affecting the function of lipid-lowering drug targets on AMI.

**Methods:**

Single-nucleotide polymorphisms (SNPs) associated with lipids as instruments were extracted from the Global Lipids Genetics Consortium (GLGC). The genome-wide association study (GWAS) data for AMI were obtained from UK Biobank. Two sample MR analysis was used to study the associations between high-density lipoprotein (HDL) cholesterol, low-density lipoprotein (LDL) cholesterol, and triglycerides (TG) with AMI (*n* = 3,927). Genetic variants associated with LDL cholesterol at or near drug target gene were used to mimic drug effects on the AMI events in drug target MR.

**Results:**

Genetically predicted higher LDL-C (per one SD increase in LDL-C of 38.67 mg/dL, OR 1.006, 95% CI 1.004–1.007) and TG (per one SD increase in TG of 90.72 mg/dL, 1.004, 1.002–1.006) was associated with increased risk of AMI, but decreased risk for higher HDL-C (per one SD increase in HDL-C of 15.51 mg/dL, 0.997, 0.995–0.999) in univariable MR. Association remained significant for LDL-C, but attenuated toward the null for HDL-C and TG in multivariable MR. Genetically proxied lower LDL-C with genetic variants at or near the *PCSK9* region (drug target of evolocumab) and *NPC1L1* (drug target of ezetimibe) were associated with decreased risk of AMI (0.997, 0.994–0.999 and 0.986, 0.975–0.998, respectively), whereas genetic variants at *HMGCR* region (drug target of statin) showed marginal association with AMI (0.995, 0.990-1.000). After excluding drug target-related SNPs, LDL-C related SNPs outside the drug target region remained a causal effect on AMI (0.994, 0.993–0.996).

**Conclusions:**

The findings suggest that genetically predicted LDL-C may play a predominant role in the development of AMI. The drug MR results imply that ezetimibe and evolocumab may decrease the risk of AMI due to their LDL-C lowering effect, and there are other non-drug related lipid lowering pathways that may be causally linked to AMI.

**Supplementary Information:**

The online version contains supplementary material available at 10.1186/s12944-024-02133-w.

## Introduction

Coronary artery disease (CAD) is characterized by the presence of atherosclerosis in coronary arteries and the leading cause of mortality and loss of disability-adjusted life-years globally [1, 2]. As one of the most severe manifestations of CAD, acute myocardial infarction (AMI) affects nearly three million people worldwide [3]. Lipids have been recognized as one of the most important modifiable risk factors for AMI as demonstrated in observational studies and randomized controlled trials (RCTs) [4–6]. Thus, investigation on primary prevention in population at risk for AMI through lipid lowering therapy may help address whether these associations are explained by a direct effect of lipid lowering or a potential specific mechanism in response to drug treatment.

Statins (3-hydroxy-3-methygutaryl coenzyme A reductase inhibitors), the lipid lowering drug, has shown effect in reducing cardiovascular events and slows disease progression among patients who has had coronary diseases in a RCT [7]. Besides clinical evidence of the benefits of statin therapy on major vascular events including AMI [8, 9], genetic evidences of drug effects of statin on -cardiovascular disease (CVD) are also emerging [10, 11]. Studies showed that naturally randomly allocated genetic variants to lower low density lipoprotein cholesterol (LDL-C) level in the *HMGCR* gene (target of statins) were associated with a lower risk of coronary events [10]. In addition, other drugs that modulate LDL-C levels such as ezetimibe and evolocumab were also evaluated for the effect on CVD [11]. However, the drug effect of lipid lowering on AMI has not been thoroughly investigated.

Using naturally occurring genetic variants as instrumental variables (IV), Mendelian randomization (MR) is able to estimate the causal effect of an exposure on the outcome and avoid limitations in observational studies such as confounding and reverse causation, since genetic variants are randomly allocated and fixed at conception. Drug-target MR is an extension of MR that offers a way to examine the direct effect of the drug on the disease outcome, in which the IVs are in the drug target region to proxy modulation of the drug target [12, 13]. When multiple exposures are present, multivariable MR can be used to estimate the direct causal effect of each exposure with adjustment of other exposures [14, 15].

Therefore, in the present study, we first performed a two-sample drug-target MR analysis to examine the associations between lipids (high density lipoprotein cholesterol (HDL-C), LDL-C, and triglycerides (TG)) and AMI using summary statistics from large genome-wide association studies (GWAS). We further explored the effects of lipid management on AMI by selecting IVs at or near the *HMGCR* (drug target of statin), *PCSK9* (drug target of evolocumab), and *NPC1L1* (drug target of ezetimibe) gene regions to investigate the lipid-lowering drug treatment on AMI and explore whether these associations are explained by a direct effect of or independent of lipid lowering.

## Methods

### Study design

**Fig. 1:**
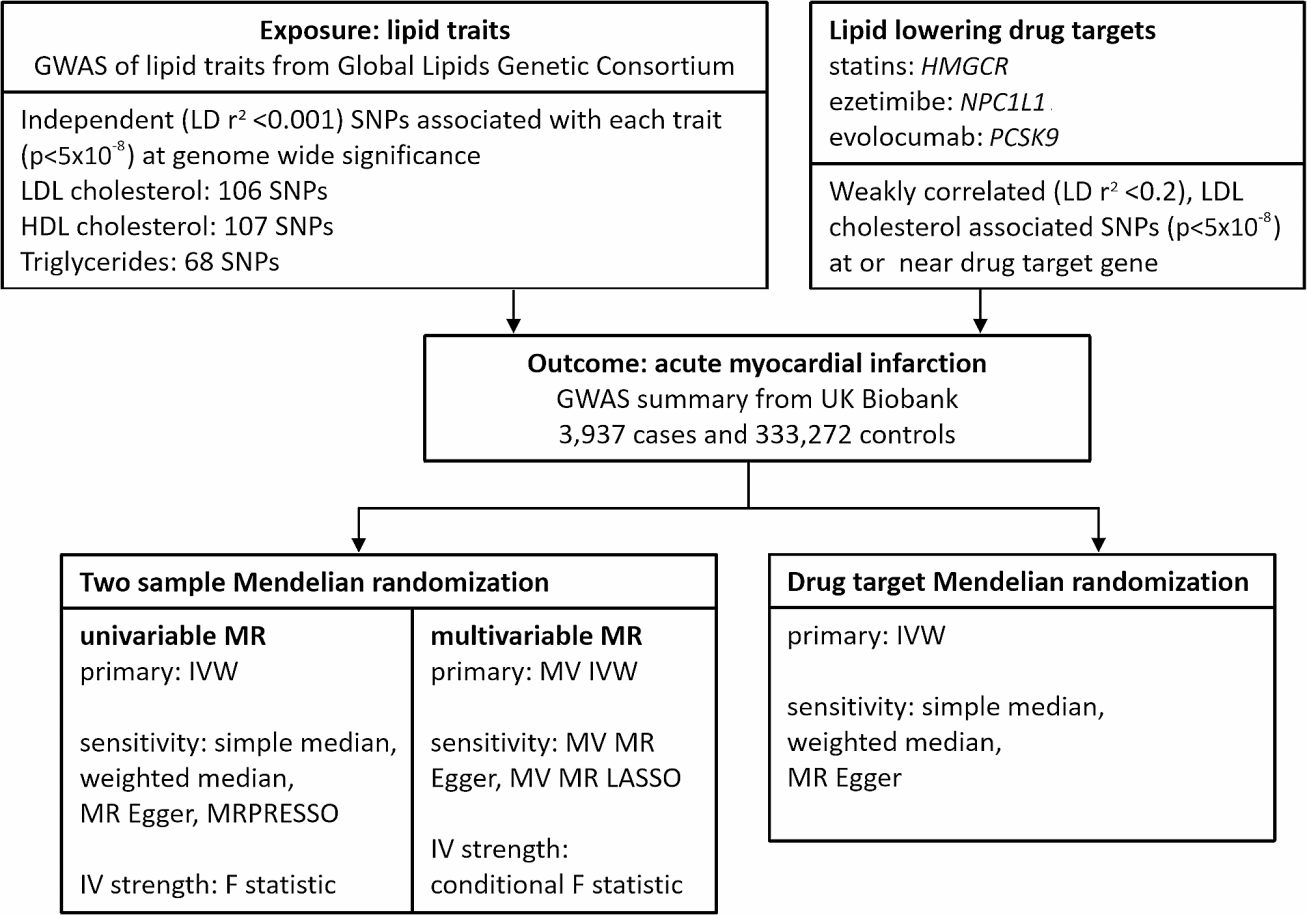
Study design

Figure 1 depicts the study design. Both univariable and multivariable MR were performed to analyze the overall and adjusted effects of different lipids on AMI. The drug-target MR was used to investigate the effects of lipid-lowering drugs on AMI. The difference between drug-target MR and conventional MR is that drug-target MR studies the direct causal effect of drug perturbation, whereas the conventional MR investigates the causal effect between exposure and outcome in general. There are also difference in genetic instrument selection process, which we will entail in the following section.

### Data materials

Summary statistics of GWAS for AMI were obtained from the UK Biobank (3,927 cases, 333,272 controls). AMI was identified using the ICD-10 code I21. Briefly, UK Biobank provides AMI GWAS summary data obtained from around 500,000 people of European descent in the United Kingdom. All detailed genotyping, quality control and imputation procedures are described on the UK Biobank website. Informed consents were obtained from all participants. Summary GWAS data of blood lipids were extracted from the Global Lipids Genetics Consortium (GLGC) [16].

### Instrumental variable selection

In order to generate genetic instruments for each lipid, independent SNPs associated with lipid level at the genome-wide significant level (linkage disequilibrium r^2^ < 0.001, *p* < 5 × 10^− 8^) were selected. For multivariable MR, all genome-wide significant SNPs across lipids were pooled out for each trait. The SNPs were then removed from analysis if they were related to blood pressure or hypertension by checking the PhenoScanner database [17]. In summary, 107 variants were associated with LDL-C, 106 with HDL-C, and 68 with triglycerides were used to proxy lipid levels. We then applied the MR Steiger filtering on SNPs and all of them showed a greater correlation with the exposure compared to the outcome [18], indicating the causal direction that pointed from exposure to the outcome.

For drug-target MR analysis, genetic variants were selected as written in guidelines and previous research [12, 13, 19] to reflect pharmacological perturbation of the drug based on associations with biomarkers. Basically, SNPs that reached a genome-wide significant level (*p* < 5 × 10^− 8^) with LDL-C at or near the *HMGCR*, *PCSK9*, and *NPC1L1* gene regions (within +-100 kb windows) were obtained to proxy lipid-lowering effects of statins, evolocumab, and ezetimibe. The selected SNPs were clumped to be in weak LD (r^2^ < 0.2 for *HMGCR*/*PCSK9*, r^2^ < 0.3 for *NPC1L1*) with other SNPs so that at least 3 SNPs were included to improve the instrument strength and to perform sensitivity analyses. Steiger filtering step was also applied to ensure the directionality was correct. In summary, 5 SNPs in *HMGCR*, 11 SNPs in *PCSK9*, and 3 SNPs in *NPC1L1* region were used as IVs in drug-target MR analysis.

### Statistical analysis

There are three assumptions for genetic instruments to be valid in MR analysis: (1) instruments are associated with the exposure, (2) affect the outcome via the exposure only, (3) and are not associated with confounders in the exposure-outcome association [13]. We estimated the proportion of phenotypic variance explained by the instrument and the F statistics. F statistics larger than 10 were accepted as evidence against weak instrument bias by convention [20].

Inverse-variance weighted (IVW) method was used as the primary MR method in the analysis and we reported the estimates of the causal effect of one SD increase in the genetically proxied exposure on the outcome [21, 22]. In multivariable MR analysis, the multivariable IVW methods were used with lipid traits including HDL-C, LDL-C, and TG to investigate the independent effects of lipids, taking into account the potential pleiotropic effect of other lipids.

Finally, we tested the genetic effects of common lipid-lowering drugs and studied if the drug effect is independent of general lipid lowering in the body using drug-target MR. Drug-target MR uses SNPs at or near the drug target as IVs to proxy drug effects on lipids and examines the effect of the drug on the AMI via lipids. We studied 3 drug effects on AMI, namely statin, ezetimibe, and evolocumab, using LDL-C related SNPs that were close to their respective drug targets *HMGCR*, *PCSK9*, and *NPC1L1* as genetic instruments. If perturbing the gene target had a significant effect on the AMI, LDL-C associated SNPs outside of the target genes were used as instruments to study whether non-drug related pathways were associated of AMI. In addition, leave-one-out analyses were performed as sensitivity analyses to test if the estimates were driven by any single SNP by re-calculating the effect estimates with one SNP removed at a time.

We also utilized several sensitivity analyses that are more robust to the pleiotropy problem in addition to the IVW method. To account for horizontal pleiotropy, the MR-Egger method was used to estimate asymptotically unbiased causal effects with an intercept that reflected the average pleiotropic effect across genetic instruments [23]. Simple median and weighted median methods were used as additional analyses on causal effect estimation in case of pleiotropy [24]. To test for heterogeneity, the Cochran Q test statistics were calculated [25]. We further included MR-PRESSO and MR-Lasso methods in the sensitivity analysis [26, 27], and these two methods were designed to reduce heterogeneity in analysis by excluding SNPs whose causal estimates differed substantially from those of other variants.

To control for type I error rate in multiple testing [28], Bonferroni corrections were applied to the following MR analyses: univariable analysis of lipids on AMI (Bonferroni threshold of *p* < 0.017 calculated as 0.05/3 to account for 3 lipids tested in analysis) and univariable analysis of lipid-lowering drugs on AMI (Bonferroni threshold of *p* < 0.013 calculated as 0.05/4 to account for 3 drug targets and non-drug related SNPs). All statistical analyses were conducted in the statistical program R (version 4.1.3) using packages ‘twoSampleMR’ (version 0.5.8), ‘MendelianRandomization’ (version 0.9.0), and ‘MRPRESSO’ (version 1.0) [26, 29–31].

## Results

### Causal effects of plasma lipids on AMI

The IVs used in univariate MR analysis are summarized and provided in Supplementary Table 1. In univariate MR, F statistic for each SNP was larger than 10 and the Steiger test showed the direction of effect was from lipid to AMI for all SNPs.

The results from the univariate MR analysis are summarized in Table 1. The results showed that all lipids were causally associated with AMI. Per one SD increase in HDL-C, LDL-C, and TG, the odds ratios for AMI were 0.997 (95% CI, 0.995–0.999), 1.006 (95% CI, 1.004–1.007), and 1.004 (95% CI, 1.002–1.006), respectively. For LDL-C, results using simple median, weighted median, and MR-Egger methods showed consistency with results of IVW. Horizontal pleiotropy was tested with MR-Egger intercept and pleiotropic effect was observed for both HDL-C and TG with intercepts that significantly deviated from zero (Supplementary Table 3, Egger intercept *p* < 0.05). The results were consistent with funnel plots (Supplementary Fig. 1) which also showed asymmetry for HDL-C and TG. Heterogeneity was observed for HDL-C, LDL-C, and TG (Supplementary Table 4, Cochran Q test *p* < 0.05) and an additional scatter plot (Supplementary Fig. 1) also showed outliers which might influence the estimates obtained by IVW and Egger. We then performed MR-PRESSO test with removal of outliers of high heterogeneity and the results were consistent with IVW (MR-PRESSO estimate: OR 0.998, 95% CI 0.996-1.000 for HDL-C; OR 1.005, 95% CI 1.004–1.007 for LDL-C; OR 1.004, 95% CI 1.002–1.006 for TG).

**Table 1:** Causal estimates of lipid traits on acute myocardial infarction using univariable MR.

Exposure	Methods	nsnp	OR (95% CI)	*p* Value
HDL Cholesterol	Simple median	106	0.996 (0.994,0.999)	9.46E-3
	Weighted median	106	1.000 (0.998,1.003)	0.797
	MR-Egger	106	1.002 (0.999,1.006)	0.211
	Inverse variance weighted	106	0.997 (0.995,0.999)	4.95E-3
	MRPRESSO	101	0.998 (0.996,1.000)	0.015
LDL Cholesterol	Simple median	107	1.005 (1.003,1.007)	7.52E-7
	Weighted median	107	1.005 (1.004,1.007)	1.20E-9
	MR-Egger	107	1.006 (1.004,1.008)	5.89E-8
	Inverse variance weighted	107	1.006 (1.004,1.007)	2.71E-15
	MRPRESSO	105	1.005 (1.004,1.007)	1.67E-13
Triglycerides	Simple median	68	1.006 (1.003,1.009)	1.27E-4
	Weighted median	68	1.002 (1.000,1.005)	0.102
	MR-Egger	68	1.001 (0.998,1.004)	0.638
	Inverse variance weighted	68	1.004 (1.002,1.006)	8.90E-5
	MRPRESSO	67	1.004 (1.002,1.006)	6.66E-5

OR: odds ratio; CI: confidence interval; MR-Egger: Mendelian randomization Egger method.

Causal estimates were shown using SNPs associated with each lipid traits, using univariable MR analysis. ORs represent the risk of the outcome per SD increase in the exposure.

In multivariable MR, conditional F statistics were calculated for each lipid and were larger than 10, showing evidence against weak instrument bias by convention (Supplementary Table 5). Multivariable MR showed that causal effect of LDL-C on AMI (OR 1.005, 95% CI 1.003–1.006) was independent of HDL-C and TG (Table 2). The estimated causal effect of LDL-C was comparable to that in the univariate MR analysis. In contrast, the associations of genetically predicted HDL-C and TG with risk of AMI did not reach statistical significance (MVMR IVW estimate: OR 0.999, 95% CI 0.997–1.001 for HDL-C; OR 1.002, 95% CI 1.000-1.004 for TG). Instrumental heterogeneity was observed in multivariable MR analysis (Supplementary Table 5, Cochran Q test *p* < 0.05) and after removing SNPs with high heterogeneity, MR-Lasso showed that causal estimate was consistent for LDL-C with IVW (MR- Lasso estimate: OR 1.005, 95% CI 1.004–1.006). MR Egger showed that the intercept differed significantly from zero and indicated a pleiotropic effect for SNPs (Supplementary Table 3, Egger intercept *p* < 0.05), but the causal estimate was comparable to the result in IVW analysis on LDL-C and AMI (MR Egger estimate: OR 1.005, 95% CI 1.003–1.006).

**Table 2:** Causal estimates of lipid traits on acute myocardial infarction using multivariable MR.

Methods	Exposure	nsnp	OR (95% CI)	*p* Value
MR IVW	HDL Cholesterol	258	0.999 (0.997,1.001)	0.179
	LDL Cholesterol	258	1.005 (1.003,1.006)	4.18E-10
	Triglycerides	258	1.002 (1.000,1.004)	0.090
MR Egger	HDL Cholesterol	258	1.002 (0.999,1.004)	0.125
	LDL Cholesterol	258	1.005 (1.003,1.006)	6.55E-11
	Triglycerides	258	1.001 (0.999,1.003)	0.229
MR-Lasso	HDL Cholesterol	228	0.999 (0.997,1.000)	0.168
	LDL Cholesterol	228	1.005 (1.004,1.006)	3.28E-18
	Triglycerides	228	1.002 (1.000,1.003)	0.029

OR: odds ratio; CI: confidence interval; MR-Egger: Mendelian randomization Egger method; MR-Lasso: Mendelian randomization Lasso method.

Causal estimates were shown for each lipid, using multivariable MR to account for effects of other lipid traits. ORs represent the risk of the outcome per SD increase in the exposure, taking other lipids into consideration.

### Impact of lipid-lowering drugs on AMI

**Fig. 2:**
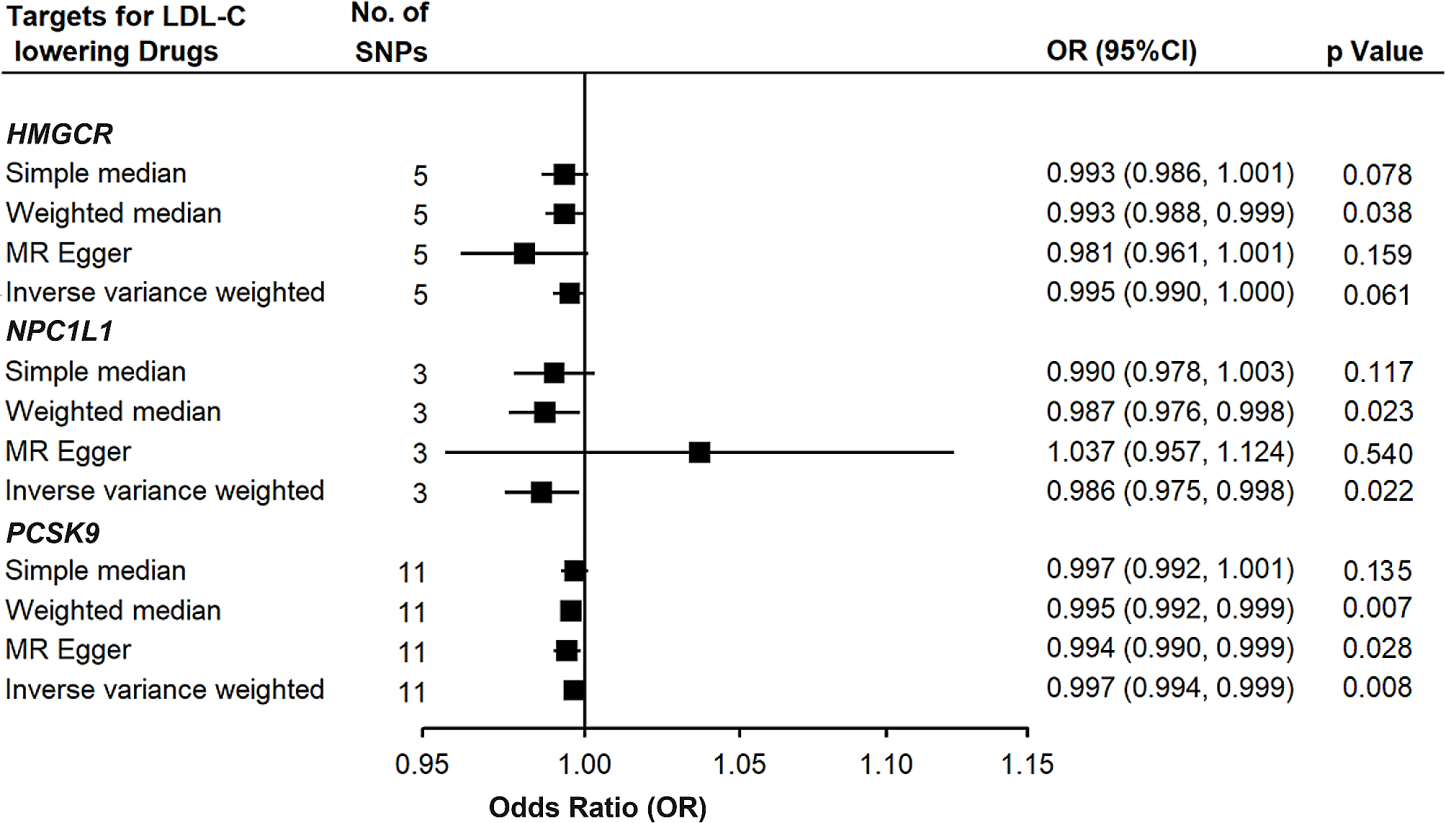
Results of association between lowing drugs and CAD

Details of the genetic variants used in drug-target MR are shown in Supplementary Table 2. The estimated causal effects of LDL-C levels using SNPs on each drug target region on AMI are displayed in Fig. 2. Results with 3 SNPs in the *NPC1L1* region showed LDL-C decrease due to ezetimibe had a causal effect on AMI (OR 0.986, 95% CI 0.975–0.998) and LDL-C proxied by 11 SNPs in the *PCSK9* region (mimicking the effect of evolocumab) had a causal effect of 0.997 (95% CI 0.994–0.999). However, LDL-C decrease predicted by genetic variations in *HMGCR* region (mimicking the effect of statin) displayed a marginal causal effect on the AMI (OR 0.995, 95% CI 0.990-1.000, *p* = 0.061). Since lipid management nowadays usually involves a combination of lipid lowering drugs, we estimated the causal effect using a combination of drugs and the results suggested a clear effect on the AMI when either two out of the three drugs were used together (IVW estimates: OR 0.993, 95% CI 0.988–0.998 for *HMGCR* plus *NPC1L1*; OR 0.996, 95% CI 0.993–0.999 for *NPC1L1* plus *PCSK9*; OR 0.996, 95% CI 0.994–0.999 for *PCSK9* plus *HMGCR*, Supplementary Table 7). Cochran Q test showed no evidence of heterogeneity in SNPs at or near the drug target regions. For *PCSK9*, single SNP forest plots showed the causal effect estimates for SNPs were of different directions (Supplementary Fig. 2), but the confidence intervals surrounding the estimates were wide and overlapped. The additional leave-one-out sensitivity analysis showed comparable-results for each SNP and all SNPs displayed inverse associations with the outcome, indicating that associations were not driven by any individual SNPs (Supplementary Fig. 2). There was no horizontal pleiotropic effect detected when performing drug-target MR using the Egger method (Supplementary Table 6, Egger intercept *p* > 0.05).

We also analyzed the biomarkers downstream of the drug target, namely LDL-C, and qualitatively assessed whether LDL-C mediated the effect of perturbing the drug target and AMI. In order to obtain effect of downstream LDL-C on AMI, we genetically proxied LDL-C level using genome-wide significant SNPs but excluded those SNPs from *HMGCR*, *NPC1L1*, and *PCSK9* gene regions. A total of 104 non-drug target SNPs for LDL-C were used and the results showed a significant causal effect of LDL-C on AMI (IVW estimator: OR 0.994, 95% CI 0.993–0.996, Table 3). The results showed that LDL-C acted as a mediator and at least part of the drug effect on AMI is mediated through LDL-C. Taken together the drug target MR results and the mediation results, we conclude that there is evidence that the three lipid lowering treatments may reduce the risk of AMI by their effect on lipid lowering medicated through LDL-C.

**Table 3:** Causal estimates of lower LDL-C proxied by drug target SNPs on AMI.

Target gene (drug)	nsnp	Method	OR (95% CI)	*p* Value
LDL-C related SNPs without drug target SNPs	104	Simple median	0.996 (0.994,0.998)	3.28E-5
	104	Weighted median	0.995 (0.993,0.997)	3.83E-8
	104	MR-Egger	0.994 (0.991,0.996)	3.64E-7
	104	IVW	0.994 (0.993,0.996)	1.76E-13

SNP: single nucleotide polymorphism; LDL-C: low density lipoprotein cholesterol; MR-Egger: Mendelian randomization Egger method; IVW: inverse variance weighted;

nsnp: number of SNPs used in analysis; OR: odds ratio; CI: confidence interval

## Discussion

Our study utilized two-sample MR approaches and showed a positive association between LDL-C and AMI. Furthermore, the drug-target MR demonstrated a causal relationship of variants at *PCSK9* (proxies for evolocumab) and *NPC1L1* (proxies for ezetimibe) region, but not *HMGCR* region serving as proxies for statins, with AMI. Taken together, our findings suggest that the effects of ezetimibe and evolocumab on risk of AMI are at least partly due to lowering LDL-C. In the case of statin, although a causal relationship was not found using the GWAS data, our findings indicate a direct effect of the drug target on AMI independent of the lipid lowering pathway, and may be informative for future applications in clinical practice.

Our results showed that LDL-C had direct causal effects on AMI, regardless of other lipid fractions using univariate and multivariable MR. The results were in consistence with a large prospective study in European and North America population [32], with an estimated hazard ratio (HR) of 1.38 (95% CI 1.09–1.73) per SD increase in LDL-C. A recent study of China Kadoorie Biobank including 912 MI patients also showed LDL-C had a causal effect on MI, stratified by different LDL-C particle sizes [6].

In univariable MR analysis, we observed that higher HDL-C was causally associated with a lower risk of AMI, but the effect became attenuated on accounting for other lipid traits in multivariable MR. This finding was consistent with the result reported by Richardson et al. [33], which showed similar attenuation effect of HDL-C when assessing the etiology of coronary heart events using genetic variants of lipids and apolipoproteins [34, 35]. Indeed, several MR studies investigating the relation between HDL-C and coronary heart disease had refuted that HDL-C had a causal role [36, 37], despite the fact that a high level of HDL-C was associated with a lower risk of vascular disease in observational studies [4]. The effect of TG on AMI was similar to that of HDL-C, but the results showed inconsistency with some published papers using genetic variants associated with TG. Do et al. showed TG was causally related to the risk for coronary artery events on accounting to HDL-C and LDL-C [38]. Later Ference et al. used triglyceride lowering variants in lipoprotein lipase (LPL) gene and demonstrated a causal effect between the TG and CAD [39]. This inconsistency might be due to the complex nature of TG metabolism and the association between lipids and requires further research. In addition, the usage of different design and summary data in MR analysis might also affect the results. The use of different instrumental variables would yield effect estimates of different size using the IVW method. Some instruments might also suffer from pleiotropic effect, thus giving biased estimate.

We elucidated the effect of lipid lowering drugs on AMI and showed lifelong genetically proxied LDL-C reduction via *PCSK9* and *NPC1L1* was associated with a lower risk of AMI, which could possibly offer insights into AMI primary prevention and management for people at high risk. We disclosed evidence that lipid lowering variants in *PCSK9* and *NPC1L1* regions can reduce AMI risk by decreasing LDL-C levels. The results were in line with what Ference et al. reported in 2015 and 2016, showing that genetically proxied lower LDL-C with *PCSK9* and *NPC1L1* played a causal role in CVD [10, 11]. A more recent meta-analysis on clinical trials of evolocumab (*PCSK9* inhibitor) pooled 24 studies and showed a reduction in MI risk by 28% (OR 0.72, 95% CI 0.64–0.82) [38]. In addition, a meta-analysis on ezetimibe (targeting *NPC1L1*) found the drug had a modest cardiovascular benefit by being used either alone or with other lipid lowering drugs [40, 41]. Compared with placebo, ezetimibe alone was able to reduce the risk of MI by 13.5% (RR 0.840, 95% CI 0.801–0.934) [42]. When ezetimibe was used together with statins, the treatment would decrease the risk of non-fatal MI by 12% (RR 0.88, 95% CI 0.81–0.95) compared to treatment with only statin [39], indicating a complementary effect via different drug mechanisms. These clinical findings validate that the results were not affected by confounding genetic variants.

Though LDL-C was found to have causal risk effects on AMI, genetically proxied lower LDL-C via *HMGCR* inhibition by statins were not causally associated with AMI risk. These results seem contradictory since we had showed that drug effect of statin on AMI were partly mediated via LDL-C, but the total effect of statin on AMI was not significant on the contrary. One possible explanation is that statin may have direct effect on AMI, independent of its lipid lowering pathway. Contestably, statins might exert distinct, adverse effects on AMI, counteracting the protective effects due to lowered LDL-C levels. Regarding the mechanics, it has been suggested that statins had both LDL-C dependent and LDL-C independent effects when used for primary and secondary preventions of coronary events [43]. For instance, statins have anti-inflammatory effects and had non LDL-C effects on atherosclerosis, which is a chronic inflammatory process, by reducing inflammatory cytokines [44]. Although our two-sample MR results showed no significant effect of LDL-C lowering with genetic variants in *HMGCR* on AMI, there is the possibility that statins might exert effects via the LDL-C independent pathways. However, Ference et al. showered the effect of LDL-C lowering on MI mediated by genetic variants in *HMGCR* was significant using one sample MR analysis in 14 pooled cohorts [10]. Further research might follow this thread and see if the results were reproducible.

The study has the following strengths. First, the analysis used genetic variants in drug target regions to proxy the drug effects of commonly used LDL-C lowering agents and found a direct causal relationship of two commonly used drugs, namely ezetimibe and evolocumab, on AMI. The use of MR methods allowed bypassing of confounding and reverse causation bias at large; the inclusion of multiple SNPs should give better IVs in analysis. In two-sample MR, the utilization of large GWAS summary data also provides sufficient power to permit discovery of robust genetic instruments in MR analysis for each lipid traits. Second, multiple methods were used to test the robustness of the estimates under different assumptions. We employed methods such as MR-Egger and MR-PRESSO. The MVMR, which takes into account of multiple exposures, allows for direct effect estimation. In addition, multiple means were taken to ensure the quality of selected IVs, such as checking the F statistic, heterogeneity, and pleiotropy. To our knowledge, we are the first study on drug effects on AMI with three lipid lowering drugs studied individually or together.

There are also several limitations. Lipid lowing drugs, such as statins, can be used in patients with AMI to prevent not only incidence but also recurrences. The limited data we used did not differentiate between the incident or recurrent AMI, which might bias the statin’s effect on the risk of AMI. Besides, the summary GWAS data of lipids and AMI were both derived from individuals primarily of European ancestry. Thus, the results should not be generalized to other populations of different ancestry. Finally, the drug target MR results need to be interpreted with caution. For one thing, genetic effects are lifelong and are often small, whereas clinical drug effects are usually short-term and larger in the magnitude of intervention. Therefore, estimates from MR should not be viewed as the equivalence of the expected effect of an intervention.

In summary, our study provided strong evidence to the causal relationship between LDL-C and AMI. Drug MR analysis further indicated that the effects of lipid-lowering drugs such as ezetimibe and evolocumab on AMI may at least partly due to their LDL-C lowering effect. Further investigations are required to explore the potential mechanisms by which drug effect prevents AMI.

### Electronic supplementary material

Below is the link to the electronic supplementary material.

Supplementary Material 1

## Data Availability

No datasets were generated or analysed during the current study.
