# Supplementary material for: Effects of genetically proxied lipid-lowering drugs on acute myocardial infarction: a drug-target mendelian randomization study
Source: Lipids Health Dis. 2024 Jun 3;23:163. doi: 10.1186/s12944-024-02133-w (PMC11145822; doi:10.1186/s12944-024-02133-w)
Supplement: Supplementary file 1 — Supplementary Material 1 [file 12944_2024_2133_MOESM1_ESM.docx]

**Supplemental materials**

**Supplementary Methods**

**Supplementary Figures**

**Supplementary Figure 1.** Scatter plots and funnel plots showing causal estimates of lipid traits on acute myocardial infarction.

**Supplementary Figure 2.** Single SNP and leave-one-out sensitivity analysis in drug-target MR for drug targets of statins (*HMGCR*), ezetimibe (*NPC1L1*), and evolocumab (*PCSK9*).

**Supplementary Tables**

**Supplementary Table 1.** Univariable MR instrumental variables for HDL-C, LDL-C, and TG extracted from Global Lipids Genetics Consortium.

**Supplementary Table 2.** Univariable MR instrumental variables for *HMGCR*, *PCSK9*, and *NPC1L1* region extracted from Global Lipids Genetics Consortium.

**Supplementary Table 3.** Cochran Q test of homogeneity in univariable and multivariable MR.

**Supplementary Table 4.** Conditional F statistics of IVs in multivariable MR analysis. **Supplementary Table 5**. Test of heterogeneity and horizontal pleiotropy for instrumental variables in drug-target MR.

**Supplementary Table 6.** Causal effect estimates using SNPs of combined lipid lowering drugs on AMI in univariable MR analysis.

**Supplementary Table 7**. Causal effect estimates of lipid lowering drugs on AMI with univariable MR analysis.

Supplementary Figure 1. Scatter plots and funnel plots showing causal estimates of lipid traits on acute myocardial infarction.


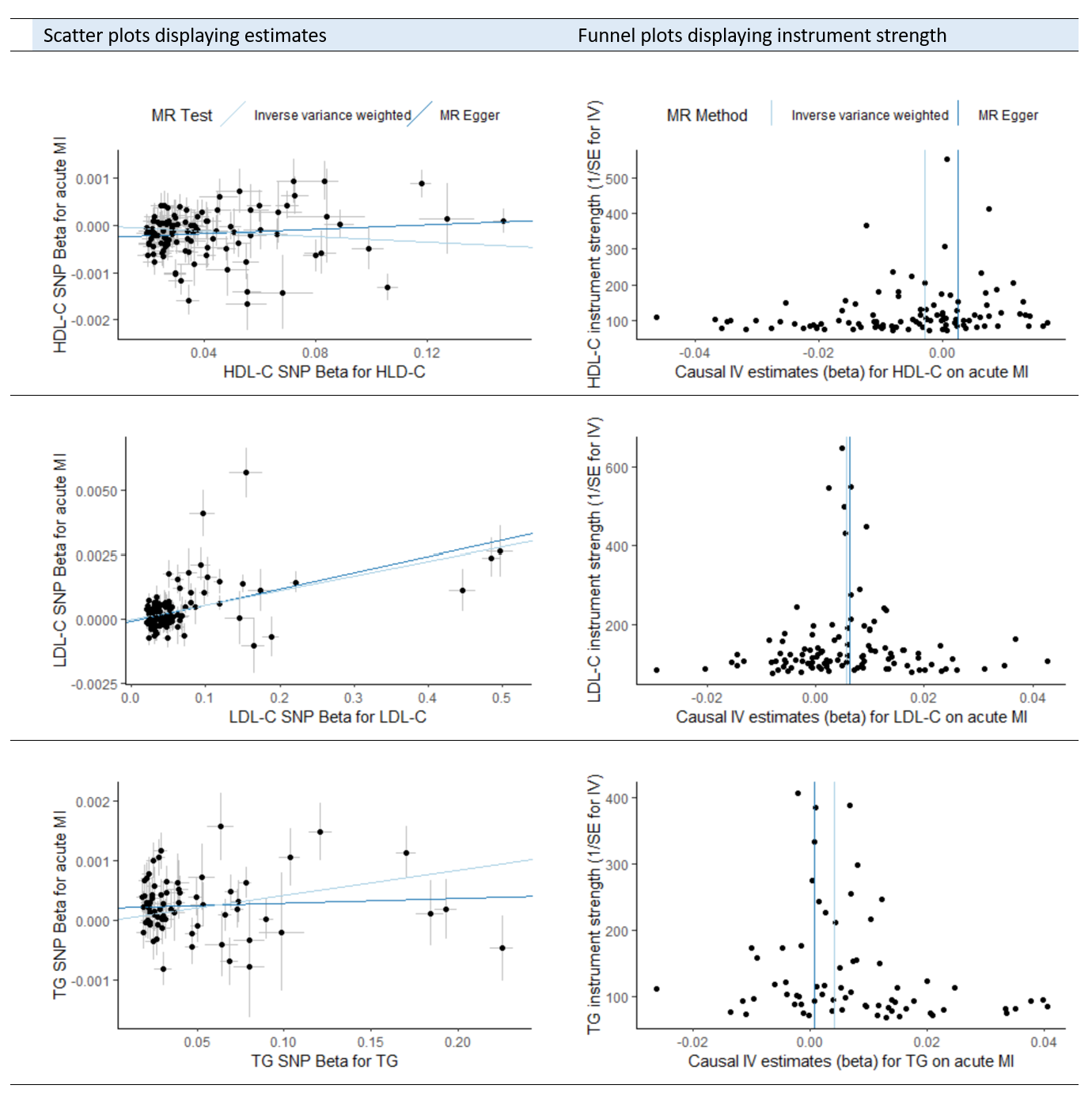


MR: Mendelian randomization; HDL-C: high density lipoprotein cholesterol; LDL-C: low density lipoprotein cholesterol; TG: triglyceride; SNP: single nucleotide polymorphism; IV: instrumental variable.

The scatterplots displayed effect estimates for each SNP associated with lipid traits. The error bar on each of the point represents 95% CI for effect of SNP-lipid (horizontal) and effect of SNP-AMI (vertical). The funnel plots showed the instrument strength against the causal effect estimates. Two MR methods, the MR Egger and the MR IVW, were showed in the plots.

Supplementary Figure 2. Single SNP and leave-one-out sensitivity analysis in drug-target MR for drug targets of statins (*HMGCR*), ezetimibe (*NPC1L1*), and evolocumab (*PCSK9*).


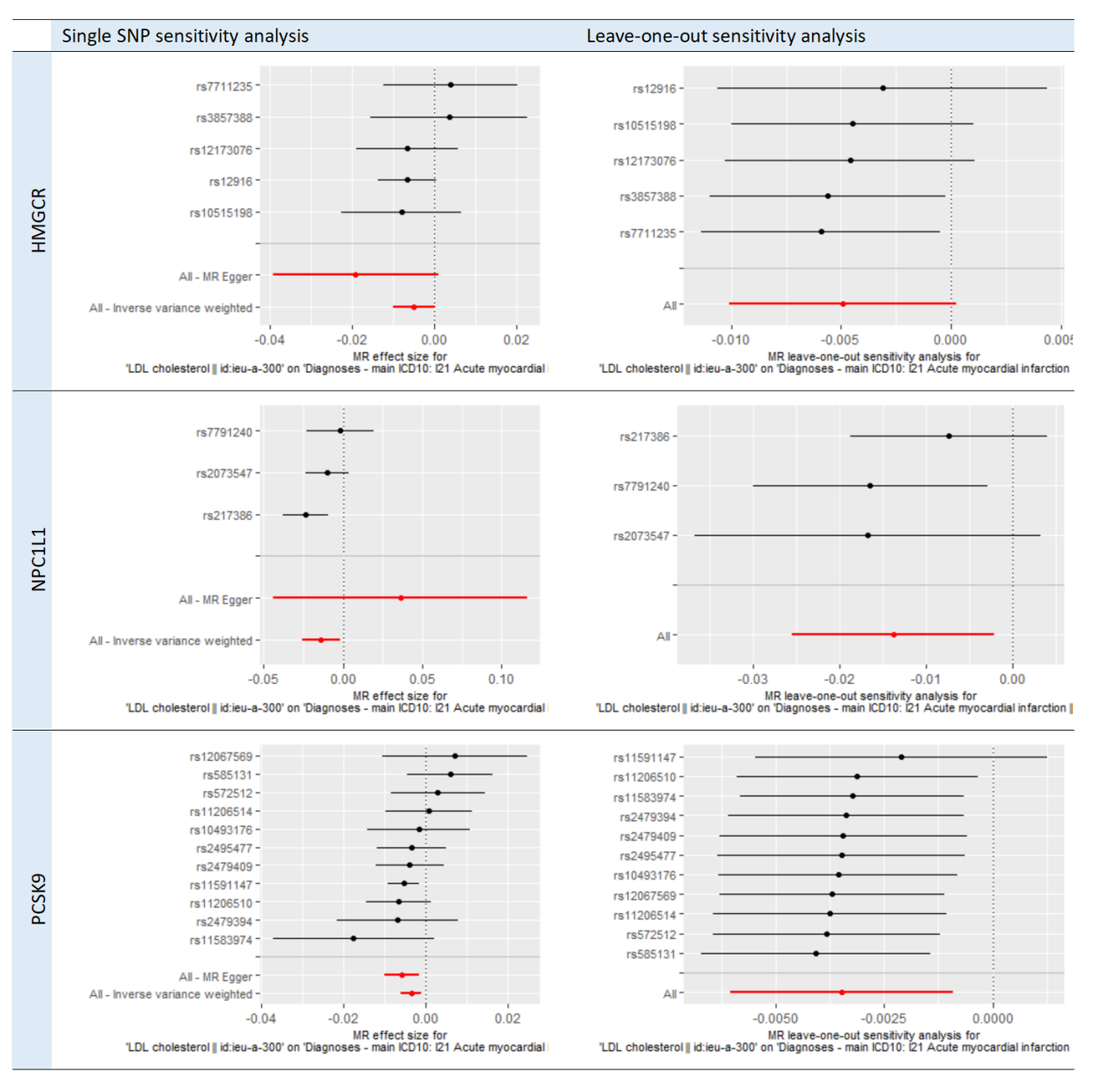


HMGCR: 3-hydroxy-3-methyglutaryl-coenzyme A receptor; NPC1L1: Niemann-Pick C1-Like 1; PCSK9: proprotein convertase subtilisin/kexin type 9; SNP: single nucleotide polymorphism.

The plots showed the effect estimates for each SNP on the outcome. The SNPs were oriented such that the lipid lowering allele was the effect allele. The overall effect of SNPs from each drug target region was summarized using the MR Egger and the MR IVW methods.

Supplementary Table 1. Univariable MR instrumental variables for HDL-C, LDL-C, and TG extracted from Global Lipids Genetics Consortium.

| Lipids&SNP | EA | NEA | EAF | Effect (beta) | Standard Error | p Value | F Statistic | R2 | Steiger direction | Steiger p value |
| --- | --- | --- | --- | --- | --- | --- | --- | --- | --- | --- |
| HDL cholesterol (nsnp=106) | | | | | | | | | | |
| rs10019888 | G | A | 0.1636 | -0.027 | 0.0046 | 4.90E-08 | 34.45 | 0.00018 | TRUE | 5.20E-05 |
| rs10087900 | A | G | 0.4393 | -0.0231 | 0.0036 | 2.17E-09 | 41.17 | 0.00022 | TRUE | 3.27E-06 |
| rs102275 | C | T | 0.372 | -0.0391 | 0.0035 | 6.40E-28 | 124.80 | 0.00067 | TRUE | 6.10E-17 |
| rs103294 | T | C | 0.186 | 0.0523 | 0.0044 | 3.99E-30 | 141.29 | 0.00080 | TRUE | 4.68E-19 |
| rs10468017 | T | C | 0.2757 | 0.1179 | 0.0038 | 1.21E-188 | 962.63 | 0.00528 | TRUE | 6.64E-119 |
| rs10773003 | A | G | 0.08839 | 0.0431 | 0.0056 | 1.46E-13 | 59.24 | 0.00032 | TRUE | 7.35E-09 |
| rs10808546 | T | C | 0.4459 | 0.0409 | 0.0034 | 4.11E-30 | 144.71 | 0.00078 | TRUE | 2.33E-16 |
| rs11045163 | G | A | 0.4063 | 0.0217 | 0.0035 | 3.20E-09 | 38.44 | 0.00021 | TRUE | 7.13E-05 |
| rs11057405 | A | G | 0.09631 | -0.0412 | 0.0062 | 1.23E-09 | 44.16 | 0.00024 | TRUE | 2.31E-06 |
| rs11789603 | T | C | 0.08971 | 0.06 | 0.006 | 3.69E-21 | 100.00 | 0.00054 | TRUE | 2.56E-15 |
| rs12038699 | G | A | 0.6425 | -0.022 | 0.0035 | 3.98E-09 | 39.51 | 0.00021 | TRUE | 2.92E-06 |
| rs12133576 | G | A | 0.6451 | -0.0243 | 0.0035 | 6.15E-11 | 48.20 | 0.00026 | TRUE | 4.83E-08 |
| rs12145743 | G | T | 0.3311 | 0.0203 | 0.0036 | 1.80E-08 | 31.80 | 0.00018 | TRUE | 1.66E-05 |
| rs12412743 | T | C | 0.153 | -0.0291 | 0.0045 | 1.31E-09 | 41.82 | 0.00022 | TRUE | 1.23E-06 |
| rs12740374 | T | G | 0.2124 | 0.0343 | 0.0041 | 1.69E-15 | 69.99 | 0.00037 | TRUE | 0.000223286 |
| rs12748152 | T | C | 0.07124 | -0.0506 | 0.0062 | 9.74E-16 | 66.61 | 0.00036 | TRUE | 1.85E-10 |
| rs13076253 | C | A | 0.1478 | -0.0283 | 0.0048 | 4.96E-09 | 34.76 | 0.00019 | TRUE | 2.26E-06 |
| rs13326165 | G | A | 0.8127 | -0.0289 | 0.0043 | 9.04E-11 | 45.17 | 0.00024 | TRUE | 2.20E-07 |
| rs13702 | C | T | 0.3127 | 0.1058 | 0.0038 | 1.28E-160 | 775.18 | 0.00413 | TRUE | 1.54E-85 |
| rs1689797 | A | C | 0.3021 | -0.0358 | 0.0036 | 2.85E-21 | 98.89 | 0.00053 | TRUE | 1.48E-14 |
| rs16942887 | A | G | 0.1332 | 0.0831 | 0.0051 | 8.28E-54 | 265.50 | 0.00143 | TRUE | 1.17E-31 |
| rs16965220 | A | C | 0.2982 | 0.0219 | 0.0037 | 7.91E-09 | 35.03 | 0.00019 | TRUE | 4.60E-06 |
| rs17120244 | T | C | 0.1544 | 0.0443 | 0.0045 | 1.17E-21 | 96.91 | 0.00052 | TRUE | 1.58E-14 |
| rs17135399 | G | A | 0.06596 | -0.0483 | 0.0077 | 4.26E-09 | 39.35 | 0.00021 | TRUE | 6.28E-05 |
| rs17145738 | T | C | 0.1174 | 0.0408 | 0.0053 | 4.95E-13 | 59.26 | 0.00032 | TRUE | 1.39E-09 |
| rs17173637 | C | T | 0.09763 | -0.0363 | 0.0057 | 1.90E-08 | 40.56 | 0.00022 | TRUE | 4.50E-05 |
| rs17695224 | A | G | 0.2388 | -0.029 | 0.0039 | 2.42E-13 | 55.29 | 0.00030 | TRUE | 2.60E-09 |
| rs1787328 | C | T | 0.4617 | 0.0411 | 0.0037 | 2.30E-26 | 123.39 | 0.00068 | TRUE | 2.65E-18 |
| rs1800961 | T | C | 0.0343 | -0.127 | 0.0099 | 1.64E-34 | 164.56 | 0.00104 | TRUE | 1.08E-25 |
| rs181360 | G | T | 0.1992 | -0.0376 | 0.0042 | 9.24E-18 | 80.15 | 0.00045 | TRUE | 5.28E-12 |
| rs1866956 | T | C | 0.6755 | 0.0217 | 0.0037 | 7.96E-10 | 34.40 | 0.00018 | TRUE | 0.000621172 |
| rs1877031 | A | G | 0.6755 | 0.0336 | 0.0036 | 1.20E-19 | 87.11 | 0.00047 | TRUE | 9.10E-12 |
| rs1883025 | T | C | 0.2427 | -0.0698 | 0.0041 | 1.50E-65 | 289.83 | 0.00155 | TRUE | 1.49E-37 |
| rs1936800 | T | C | 0.4723 | -0.02 | 0.0034 | 3.05E-10 | 34.60 | 0.00018 | TRUE | 0.000155188 |
| rs2013208 | T | C | 0.5053 | 0.0254 | 0.0036 | 8.92E-12 | 49.78 | 0.00029 | TRUE | 1.90E-05 |
| rs205262 | G | A | 0.2665 | -0.0283 | 0.0039 | 3.88E-13 | 52.66 | 0.00029 | TRUE | 4.84E-08 |
| rs2066714 | C | T | 0.1201 | 0.0453 | 0.0071 | 7.26E-10 | 40.71 | 0.00044 | TRUE | 8.21E-07 |
| rs2075650 | G | A | 0.1266 | -0.0554 | 0.0051 | 9.72E-26 | 118.00 | 0.00067 | TRUE | 4.65E-11 |
| rs2233455 | T | C | 0.06464 | 0.0567 | 0.0066 | 2.27E-14 | 73.80 | 0.00040 | TRUE | 7.19E-11 |
| rs2241210 | G | A | 0.5528 | 0.0332 | 0.0035 | 2.49E-20 | 89.98 | 0.00051 | TRUE | 4.81E-14 |
| rs2241770 | C | T | 0.1029 | -0.0989 | 0.0057 | 6.78E-60 | 301.05 | 0.00162 | TRUE | 3.89E-40 |
| rs2250802 | A | G | 0.6807 | -0.034 | 0.0038 | 2.02E-17 | 80.06 | 0.00043 | TRUE | 3.94E-12 |
| rs2278236 | A | G | 0.5435 | 0.0331 | 0.0035 | 3.18E-18 | 89.44 | 0.00048 | TRUE | 2.02E-10 |
| rs2288911 | G | T | 0.5053 | 0.0302 | 0.0036 | 2.84E-15 | 70.37 | 0.00039 | TRUE | 1.84E-09 |
| rs2293889 | G | T | 0.5871 | 0.0312 | 0.0035 | 4.27E-17 | 79.46 | 0.00044 | TRUE | 2.40E-10 |
| rs2412710 | A | G | 0.02243 | -0.084 | 0.0139 | 1.36E-09 | 36.52 | 0.00022 | TRUE | 1.25E-06 |
| rs2454722 | G | A | 0.1451 | 0.0351 | 0.0044 | 3.31E-14 | 63.64 | 0.00034 | TRUE | 1.60E-10 |
| rs2602836 | G | A | 0.5726 | -0.0192 | 0.0034 | 4.96E-08 | 31.89 | 0.00017 | TRUE | 9.44E-06 |
| rs2642438 | G | A | 0.7454 | 0.0303 | 0.0039 | 7.78E-14 | 60.36 | 0.00034 | TRUE | 4.04E-10 |
| rs2652840 | C | T | 0.7665 | 0.0272 | 0.0042 | 1.09E-10 | 41.94 | 0.00023 | TRUE | 2.94E-06 |
| rs2844513 | A | G | 0.4763 | 0.0233 | 0.0038 | 2.47E-09 | 37.60 | 0.00024 | TRUE | 9.57E-07 |
| rs2865620 | T | C | 0.6412 | -0.0194 | 0.0036 | 4.09E-08 | 29.04 | 0.00016 | TRUE | 6.58E-05 |
| rs333947 | A | G | 0.1464 | -0.0296 | 0.0047 | 3.17E-09 | 39.66 | 0.00022 | TRUE | 1.48E-06 |
| rs3741414 | T | C | 0.1913 | 0.0296 | 0.004 | 6.10E-14 | 54.76 | 0.00029 | TRUE | 9.54E-05 |
| rs3790106 | G | C | 0.19 | -0.0374 | 0.0052 | 3.27E-11 | 51.73 | 0.00030 | TRUE | 1.31E-08 |
| rs3800406 | G | A | 0.1135 | -0.035 | 0.0058 | 2.52E-09 | 36.41 | 0.00020 | TRUE | 1.14E-05 |
| rs3822072 | A | G | 0.4881 | -0.0251 | 0.0034 | 4.06E-12 | 54.50 | 0.00029 | TRUE | 1.46E-08 |
| rs3861397 | G | A | 0.3417 | -0.024 | 0.0036 | 8.40E-11 | 44.44 | 0.00024 | TRUE | 1.04E-07 |
| rs4142995 | T | G | 0.3839 | -0.0263 | 0.0037 | 9.36E-12 | 50.53 | 0.00031 | TRUE | 1.17E-08 |
| rs4148005 | G | T | 0.2995 | -0.0283 | 0.0036 | 5.74E-14 | 61.80 | 0.00033 | TRUE | 5.88E-10 |
| rs4240624 | A | G | 0.9248 | 0.0818 | 0.0058 | 1.32E-45 | 198.91 | 0.00107 | TRUE | 4.85E-26 |
| rs424346 | T | C | 0.04881 | 0.0679 | 0.0113 | 4.84E-08 | 36.11 | 0.00028 | TRUE | 3.22E-05 |
| rs4379922 | C | T | 0.3496 | 0.0247 | 0.0036 | 9.56E-12 | 47.07 | 0.00025 | TRUE | 2.72E-05 |
| rs442177 | T | G | 0.5528 | -0.0215 | 0.0034 | 2.19E-09 | 39.99 | 0.00021 | TRUE | 9.21E-07 |
| rs4465830 | G | A | 0.2018 | -0.0597 | 0.0044 | 5.17E-40 | 184.10 | 0.00099 | TRUE | 2.84E-24 |
| rs4650994 | A | G | 0.4828 | -0.021 | 0.0034 | 6.70E-09 | 38.15 | 0.00020 | TRUE | 1.57E-05 |
| rs4660214 | C | T | 0.1926 | -0.0366 | 0.0042 | 4.73E-17 | 75.94 | 0.00042 | TRUE | 3.43E-12 |
| rs4660293 | G | A | 0.2361 | -0.0353 | 0.004 | 2.86E-18 | 77.88 | 0.00042 | TRUE | 1.56E-12 |
| rs4795400 | T | C | 0.4723 | 0.0248 | 0.0034 | 8.74E-13 | 53.20 | 0.00029 | TRUE | 8.25E-09 |
| rs4846914 | A | G | 0.5844 | 0.0479 | 0.0034 | 3.51E-41 | 198.48 | 0.00106 | TRUE | 2.21E-24 |
| rs4917014 | G | T | 0.3404 | 0.0222 | 0.0036 | 1.03E-08 | 38.03 | 0.00020 | TRUE | 7.56E-06 |
| rs492571 | C | T | 0.04222 | -0.0663 | 0.009 | 1.27E-12 | 54.27 | 0.00031 | TRUE | 1.11E-08 |
| rs4939883 | C | T | 0.8193 | 0.0799 | 0.0045 | 1.80E-66 | 315.26 | 0.00170 | TRUE | 2.15E-39 |
| rs4969178 | G | A | 0.6266 | 0.0263 | 0.0035 | 1.53E-12 | 56.46 | 0.00030 | TRUE | 6.38E-08 |
| rs4983559 | A | G | 0.6227 | -0.0197 | 0.0036 | 9.57E-09 | 29.95 | 0.00016 | TRUE | 0.002545338 |
| rs499974 | A | C | 0.1755 | -0.0263 | 0.0044 | 1.12E-08 | 35.73 | 0.00019 | TRUE | 1.84E-06 |
| rs573455 | G | A | 0.5303 | -0.0221 | 0.0034 | 2.52E-10 | 42.25 | 0.00023 | TRUE | 1.86E-06 |
| rs633695 | G | A | 0.285 | 0.0885 | 0.0054 | 7.82E-58 | 268.60 | 0.00289 | TRUE | 2.33E-47 |
| rs6450176 | A | G | 0.2784 | -0.0254 | 0.0039 | 6.87E-10 | 42.42 | 0.00023 | TRUE | 3.48E-05 |
| rs6457796 | C | T | 0.2652 | -0.0264 | 0.0038 | 4.11E-12 | 48.27 | 0.00026 | TRUE | 1.31E-06 |
| rs6485672 | A | G | 0.4974 | -0.0246 | 0.0035 | 3.42E-10 | 49.40 | 0.00026 | TRUE | 2.68E-06 |
| rs6499137 | G | T | 0.08839 | 0.0722 | 0.0062 | 5.90E-28 | 135.61 | 0.00079 | TRUE | 7.99E-17 |
| rs6567160 | C | T | 0.2309 | -0.0257 | 0.0041 | 2.92E-09 | 39.29 | 0.00021 | TRUE | 6.48E-06 |
| rs676210 | A | G | 0.2309 | 0.066 | 0.004 | 2.34E-54 | 272.25 | 0.00145 | TRUE | 7.05E-38 |
| rs6805251 | C | T | 0.6187 | -0.02 | 0.0035 | 1.33E-08 | 32.65 | 0.00018 | TRUE | 0.000185847 |
| rs686030 | A | C | 0.8588 | 0.055 | 0.0049 | 4.29E-27 | 125.99 | 0.00067 | TRUE | 8.75E-15 |
| rs687339 | T | C | 0.7665 | -0.0316 | 0.0042 | 7.11E-13 | 56.61 | 0.00030 | TRUE | 0.000145198 |
| rs7014168 | A | G | 0.2414 | -0.0267 | 0.0041 | 9.20E-10 | 42.41 | 0.00023 | TRUE | 6.01E-06 |
| rs702485 | G | A | 0.4499 | 0.0243 | 0.0034 | 6.45E-12 | 51.08 | 0.00027 | TRUE | 1.04E-06 |
| rs7117842 | C | T | 0.3892 | 0.0272 | 0.0035 | 1.06E-14 | 60.40 | 0.00032 | TRUE | 1.62E-09 |
| rs7220650 | C | T | 0.2493 | -0.0362 | 0.0039 | 8.39E-19 | 86.16 | 0.00046 | TRUE | 9.43E-14 |
| rs7306660 | A | G | 0.3694 | -0.0345 | 0.0036 | 3.34E-19 | 91.84 | 0.00049 | TRUE | 1.11E-10 |
| rs731839 | A | G | 0.6583 | 0.022 | 0.0037 | 3.44E-09 | 35.35 | 0.00019 | TRUE | 0.002059732 |
| rs737337 | C | T | 0.0686 | -0.0565 | 0.0061 | 4.56E-17 | 85.79 | 0.00046 | TRUE | 6.38E-13 |
| rs74458891 | T | C | 0.07256 | 0.0526 | 0.0085 | 6.49E-09 | 38.29 | 0.00041 | TRUE | 2.00E-06 |
| rs7607980 | C | T | 0.1491 | 0.0447 | 0.0052 | 1.81E-15 | 73.89 | 0.00039 | TRUE | 1.54E-10 |
| rs7621025 | C | T | 0.7348 | -0.0297 | 0.004 | 2.24E-12 | 55.13 | 0.00029 | TRUE | 9.24E-05 |
| rs7973683 | A | C | 0.3615 | 0.0286 | 0.0036 | 5.26E-14 | 63.11 | 0.00034 | TRUE | 1.30E-09 |
| rs8044014 | C | T | 0.1438 | 0.0726 | 0.0049 | 9.06E-45 | 219.52 | 0.00118 | TRUE | 7.64E-28 |
| rs8044791 | C | T | 0.1306 | 0.0339 | 0.0053 | 2.14E-09 | 40.91 | 0.00022 | TRUE | 2.33E-06 |
| rs838876 | G | A | 0.6741 | -0.0493 | 0.0039 | 7.32E-33 | 159.80 | 0.00092 | TRUE | 1.33E-24 |
| rs918144 | T | C | 0.4565 | 0.0212 | 0.0035 | 6.47E-09 | 36.69 | 0.00021 | TRUE | 8.35E-06 |
| rs9457931 | G | A | 0.0686 | -0.0552 | 0.0073 | 7.30E-13 | 57.18 | 0.00033 | TRUE | 1.02E-05 |
| rs970548 | C | A | 0.277 | 0.0258 | 0.0039 | 1.71E-10 | 43.76 | 0.00023 | TRUE | 7.12E-06 |
| rs998584 | A | C | 0.5145 | -0.026 | 0.0038 | 2.27E-11 | 46.81 | 0.00025 | TRUE | 6.39E-06 |
| rs9989419 | G | A | 0.595 | 0.1473 | 0.0036 | 1.00E-200 | 1674.17 | 0.00934 | TRUE | 7.57E-237 |
| LDL cholesterol (nsnp=107) | | | | | | | | | | |
| rs10102164 | A | G | 0.1741 | 0.0316 | 0.0045 | 3.74E-11 | 49.31 | 0.00028 | TRUE | 1.82E-08 |
| rs10195252 | C | T | 0.4182 | -0.0238 | 0.0039 | 3.81E-08 | 37.24 | 0.00024 | TRUE | 5.35E-07 |
| rs10401969 | C | T | 0.07124 | -0.1184 | 0.0072 | 2.65E-54 | 270.42 | 0.00157 | TRUE | 3.25E-31 |
| rs10403668 | A | G | 0.1623 | -0.0439 | 0.0052 | 3.10E-15 | 71.27 | 0.00042 | TRUE | 6.07E-11 |
| rs10490626 | A | G | 0.07916 | -0.0508 | 0.0069 | 1.70E-12 | 54.20 | 0.00031 | TRUE | 4.62E-09 |
| rs10832962 | T | C | 0.719 | 0.032 | 0.004 | 6.62E-14 | 64.00 | 0.00037 | TRUE | 3.39E-10 |
| rs10893499 | A | G | 0.1438 | 0.0521 | 0.0053 | 3.86E-21 | 96.63 | 0.00056 | TRUE | 5.00E-15 |
| rs10903129 | G | A | 0.5369 | 0.0328 | 0.0037 | 3.03E-17 | 78.59 | 0.00046 | TRUE | 1.40E-11 |
| rs10947207 | C | T | 0.3087 | 0.0308 | 0.0043 | 2.45E-12 | 51.31 | 0.00032 | TRUE | 2.55E-06 |
| rs10947332 | A | G | 0.1319 | 0.0504 | 0.0056 | 6.97E-18 | 81.00 | 0.00048 | TRUE | 2.29E-10 |
| rs112201728 | T | C | 0.05805 | 0.0675 | 0.0104 | 8.51E-10 | 42.13 | 0.00051 | TRUE | 9.55E-09 |
| rs11485618 | G | A | 0.3087 | -0.05 | 0.0039 | 3.73E-33 | 164.37 | 0.00097 | TRUE | 5.42E-21 |
| rs11563251 | T | C | 0.1253 | 0.0345 | 0.0062 | 4.50E-08 | 30.96 | 0.00018 | TRUE | 0.001085025 |
| rs11591147 | T | G | 0.01715 | -0.497 | 0.018 | 8.57E-143 | 762.37 | 0.00975 | TRUE | 2.92E-124 |
| rs11679386 | C | T | 0.128 | 0.0452 | 0.0061 | 4.75E-14 | 54.91 | 0.00035 | TRUE | 2.74E-09 |
| rs1169288 | C | A | 0.3338 | 0.0375 | 0.004 | 6.45E-21 | 87.89 | 0.00054 | TRUE | 1.01E-10 |
| rs11709504 | C | T | 0.2018 | -0.0251 | 0.0047 | 4.60E-08 | 28.52 | 0.00016 | TRUE | 6.58E-05 |
| rs117733303 | G | A | 0.01187 | 0.1551 | 0.022 | 8.47E-11 | 49.70 | 0.00064 | TRUE | 0.00014711 |
| rs11881156 | T | C | 0.1623 | -0.0811 | 0.0049 | 1.70E-55 | 273.94 | 0.00160 | TRUE | 1.41E-31 |
| rs12066643 | T | C | 0.1187 | -0.0389 | 0.0064 | 1.06E-08 | 36.94 | 0.00022 | TRUE | 9.36E-07 |
| rs12410656 | T | C | 0.07388 | 0.0516 | 0.0083 | 3.57E-08 | 38.65 | 0.00027 | TRUE | 1.03E-06 |
| rs1250229 | C | T | 0.7889 | 0.0243 | 0.0042 | 3.13E-08 | 33.47 | 0.00019 | TRUE | 0.000950585 |
| rs12670798 | C | T | 0.2243 | 0.0344 | 0.0043 | 4.81E-14 | 64.00 | 0.00037 | TRUE | 1.58E-07 |
| rs12721109 | A | G | 0.01715 | -0.4462 | 0.0183 | 2.99E-122 | 594.51 | 0.00594 | TRUE | 1.42E-95 |
| rs12748152 | T | C | 0.07124 | 0.0499 | 0.0066 | 3.21E-12 | 57.16 | 0.00033 | TRUE | 2.25E-09 |
| rs12916 | C | T | 0.4314 | 0.0733 | 0.0038 | 7.79E-78 | 372.08 | 0.00221 | TRUE | 5.98E-49 |
| rs13206249 | A | G | 0.2164 | -0.0378 | 0.0062 | 4.53E-08 | 37.17 | 0.00043 | TRUE | 6.78E-07 |
| rs13277801 | T | C | 0.653 | -0.0338 | 0.0038 | 3.99E-17 | 79.12 | 0.00046 | TRUE | 4.63E-12 |
| rs1367117 | A | G | 0.2876 | 0.1186 | 0.004 | 9.48E-183 | 879.12 | 0.00506 | TRUE | 2.34E-114 |
| rs1386585 | T | C | 0.8074 | 0.0337 | 0.0046 | 2.21E-12 | 53.67 | 0.00031 | TRUE | 5.06E-07 |
| rs1534420 | G | A | 0.6583 | 0.0225 | 0.0039 | 3.90E-08 | 33.28 | 0.00019 | TRUE | 0.000302013 |
| rs1564348 | C | T | 0.1451 | 0.0481 | 0.005 | 2.76E-21 | 92.54 | 0.00053 | TRUE | 1.18E-11 |
| rs1594895 | C | T | 0.1821 | -0.0333 | 0.0046 | 4.42E-12 | 52.41 | 0.00031 | TRUE | 6.22E-08 |
| rs16831243 | T | C | 0.1807 | 0.0378 | 0.0055 | 9.06E-12 | 47.23 | 0.00029 | TRUE | 2.20E-08 |
| rs16891156 | C | A | 0.01847 | 0.0965 | 0.0171 | 8.23E-09 | 31.85 | 0.00035 | TRUE | 0.003035792 |
| rs16988072 | C | T | 0.0343 | 0.063 | 0.0102 | 1.53E-09 | 38.15 | 0.00022 | TRUE | 1.61E-06 |
| rs16996148 | T | G | 0.07388 | -0.0986 | 0.0067 | 1.97E-45 | 216.57 | 0.00127 | TRUE | 1.11E-26 |
| rs17404153 | T | G | 0.1438 | -0.0336 | 0.0054 | 1.83E-09 | 38.72 | 0.00022 | TRUE | 2.44E-06 |
| rs174583 | T | C | 0.3747 | -0.0522 | 0.0038 | 7.00E-41 | 188.70 | 0.00109 | TRUE | 7.98E-26 |
| rs17508045 | C | T | 0.07256 | -0.0488 | 0.0066 | 4.91E-12 | 54.67 | 0.00032 | TRUE | 4.67E-09 |
| rs1800961 | T | C | 0.0343 | -0.0685 | 0.0106 | 6.03E-10 | 41.76 | 0.00029 | TRUE | 1.07E-07 |
| rs1801689 | C | A | 0.03694 | 0.1028 | 0.0139 | 9.81E-12 | 54.70 | 0.00049 | TRUE | 9.45E-08 |
| rs1883025 | T | C | 0.2427 | -0.0296 | 0.0044 | 6.14E-11 | 45.26 | 0.00026 | TRUE | 3.45E-06 |
| rs2000999 | A | G | 0.1847 | 0.065 | 0.0046 | 4.22E-41 | 199.67 | 0.00116 | TRUE | 2.26E-30 |
| rs2030746 | T | C | 0.3984 | 0.0214 | 0.0038 | 8.60E-09 | 31.71 | 0.00018 | TRUE | 1.44E-05 |
| rs207145 | T | C | 0.8905 | 0.0495 | 0.0057 | 6.19E-18 | 75.42 | 0.00045 | TRUE | 1.19E-09 |
| rs2073547 | G | A | 0.1939 | 0.0485 | 0.0049 | 1.92E-21 | 97.97 | 0.00058 | TRUE | 3.95E-13 |
| rs2194562 | A | G | 0.09367 | 0.0457 | 0.0061 | 2.44E-11 | 56.13 | 0.00033 | TRUE | 2.75E-07 |
| rs2328223 | C | A | 0.2493 | 0.0299 | 0.005 | 5.63E-09 | 35.76 | 0.00021 | TRUE | 7.94E-06 |
| rs2419604 | G | A | 0.6821 | -0.0302 | 0.004 | 7.49E-14 | 57.00 | 0.00033 | TRUE | 6.17E-09 |
| rs247616 | T | C | 0.2929 | -0.0547 | 0.0041 | 2.57E-37 | 177.99 | 0.00104 | TRUE | 7.93E-23 |
| rs2587534 | A | G | 0.5277 | 0.0391 | 0.0037 | 8.06E-25 | 111.67 | 0.00065 | TRUE | 6.58E-16 |
| rs2642438 | G | A | 0.7454 | 0.0352 | 0.0042 | 7.32E-16 | 70.24 | 0.00042 | TRUE | 7.89E-12 |
| rs267733 | G | A | 0.1372 | -0.0331 | 0.0053 | 5.29E-09 | 39.00 | 0.00024 | TRUE | 1.39E-05 |
| rs2710642 | A | G | 0.6187 | 0.0239 | 0.0038 | 6.09E-09 | 39.56 | 0.00023 | TRUE | 0.000382642 |
| rs2737252 | A | G | 0.2559 | -0.0314 | 0.0041 | 7.04E-14 | 58.65 | 0.00034 | TRUE | 2.49E-09 |
| rs2886232 | C | T | 0.8799 | -0.0451 | 0.0064 | 3.88E-11 | 49.66 | 0.00031 | TRUE | 9.11E-09 |
| rs2965157 | C | T | 0.02111 | -0.1886 | 0.0112 | 7.29E-62 | 283.56 | 0.00166 | TRUE | 6.65E-40 |
| rs314253 | C | T | 0.3351 | -0.0242 | 0.0038 | 3.44E-10 | 40.56 | 0.00024 | TRUE | 2.11E-06 |
| rs364585 | G | A | 0.6332 | 0.0249 | 0.0038 | 4.28E-10 | 42.94 | 0.00025 | TRUE | 3.94E-07 |
| rs3757354 | T | C | 0.2098 | -0.0382 | 0.0044 | 2.09E-17 | 75.37 | 0.00044 | TRUE | 9.65E-11 |
| rs3780181 | G | A | 0.05277 | -0.0445 | 0.0074 | 1.76E-09 | 36.16 | 0.00021 | TRUE | 2.36E-05 |
| rs3798221 | T | G | 0.223 | -0.0368 | 0.0045 | 1.06E-15 | 66.88 | 0.00039 | TRUE | 7.71E-09 |
| rs3810444 | A | T | 0.05541 | -0.0624 | 0.0084 | 2.60E-12 | 55.18 | 0.00034 | TRUE | 6.41E-06 |
| rs413380 | C | T | 0.9657 | 0.0861 | 0.0098 | 7.62E-17 | 77.19 | 0.00045 | TRUE | 1.46E-11 |
| rs4142393 | T | C | 0.4512 | 0.0214 | 0.0036 | 1.35E-09 | 35.34 | 0.00021 | TRUE | 8.55E-05 |
| rs4253776 | G | A | 0.124 | 0.0311 | 0.0059 | 3.35E-08 | 27.79 | 0.00016 | TRUE | 0.000443341 |
| rs4361493 | G | A | 0.4077 | 0.0469 | 0.0038 | 1.11E-33 | 152.33 | 0.00088 | TRUE | 2.22E-23 |
| rs4530754 | A | G | 0.5818 | 0.0275 | 0.0036 | 3.58E-12 | 58.35 | 0.00034 | TRUE | 6.65E-08 |
| rs4722551 | C | T | 0.1702 | 0.0391 | 0.0049 | 3.95E-14 | 63.67 | 0.00037 | TRUE | 9.45E-10 |
| rs4847221 | C | T | 0.8958 | 0.0383 | 0.0057 | 8.57E-11 | 45.15 | 0.00026 | TRUE | 2.57E-07 |
| rs4942486 | C | T | 0.5383 | -0.0243 | 0.0037 | 2.26E-11 | 43.13 | 0.00025 | TRUE | 9.27E-06 |
| rs4970712 | C | A | 0.8061 | 0.0339 | 0.0044 | 2.46E-13 | 59.36 | 0.00034 | TRUE | 6.32E-09 |
| rs4970834 | T | C | 0.1873 | -0.1503 | 0.0047 | 1.00E-200 | 1022.64 | 0.00588 | TRUE | 1.44E-122 |
| rs4988235 | A | G | 0.5237 | -0.0278 | 0.0042 | 3.22E-11 | 43.81 | 0.00026 | TRUE | 7.81E-08 |
| rs508487 | T | C | 0.08179 | 0.0561 | 0.0085 | 1.77E-10 | 43.56 | 0.00026 | TRUE | 1.42E-07 |
| rs5763662 | T | C | 0.02507 | 0.0767 | 0.0121 | 1.19E-08 | 40.18 | 0.00025 | TRUE | 5.65E-05 |
| rs6016381 | C | T | 0.3602 | -0.0363 | 0.0038 | 6.85E-20 | 91.25 | 0.00053 | TRUE | 3.07E-12 |
| rs6065311 | C | T | 0.4604 | 0.0417 | 0.0036 | 1.66E-30 | 134.17 | 0.00078 | TRUE | 7.68E-20 |
| rs6504872 | T | C | 0.4723 | 0.0274 | 0.0037 | 3.48E-13 | 54.84 | 0.00032 | TRUE | 4.33E-08 |
| rs650985 | T | C | 0.9591 | 0.0651 | 0.0078 | 1.90E-15 | 69.66 | 0.00042 | TRUE | 4.92E-08 |
| rs6511720 | T | G | 0.09763 | -0.2209 | 0.0061 | 1.00E-200 | 1311.39 | 0.00763 | TRUE | 4.92E-165 |
| rs6544713 | C | T | 0.7058 | -0.0806 | 0.0041 | 4.84E-83 | 386.46 | 0.00223 | TRUE | 2.20E-48 |
| rs6709904 | G | A | 0.1135 | -0.055 | 0.0085 | 4.58E-10 | 41.87 | 0.00047 | TRUE | 1.32E-08 |
| rs6759321 | T | G | 0.4591 | 0.0244 | 0.0042 | 7.79E-09 | 33.75 | 0.00020 | TRUE | 3.51E-06 |
| rs6818397 | G | T | 0.5871 | -0.0224 | 0.004 | 1.68E-08 | 31.36 | 0.00018 | TRUE | 2.47E-05 |
| rs6882076 | C | T | 0.6662 | 0.0456 | 0.0038 | 3.31E-31 | 144.00 | 0.00083 | TRUE | 1.11E-20 |
| rs6909746 | T | C | 0.3918 | -0.0263 | 0.0037 | 7.86E-11 | 50.53 | 0.00030 | TRUE | 1.23E-08 |
| rs7188 | C | A | 0.3259 | 0.0521 | 0.0043 | 9.39E-31 | 146.80 | 0.00101 | TRUE | 7.63E-20 |
| rs7254892 | A | G | 0.03166 | -0.4853 | 0.0119 | 1.00E-200 | 1663.13 | 0.01181 | TRUE | 8.09E-232 |
| rs7255743 | A | G | 0.02639 | -0.1647 | 0.0156 | 1.41E-25 | 111.46 | 0.00090 | TRUE | 1.47E-17 |
| rs7264396 | T | C | 0.219 | -0.0246 | 0.0045 | 4.41E-08 | 29.88 | 0.00017 | TRUE | 1.46E-05 |
| rs72902576 | G | T | 0.03694 | -0.0933 | 0.0133 | 9.58E-12 | 49.21 | 0.00060 | TRUE | 1.51E-06 |
| rs73045960 | G | A | 0.02243 | -0.1456 | 0.0192 | 4.16E-12 | 57.51 | 0.00075 | TRUE | 9.44E-12 |
| rs7512480 | T | C | 0.4802 | 0.0221 | 0.0037 | 2.62E-08 | 35.68 | 0.00021 | TRUE | 0.000107411 |
| rs7551981 | T | G | 0.595 | 0.0472 | 0.0038 | 1.36E-33 | 154.28 | 0.00089 | TRUE | 1.69E-21 |
| rs75687619 | T | G | 0.02375 | 0.1735 | 0.0161 | 8.05E-24 | 116.13 | 0.00141 | TRUE | 1.32E-19 |
| rs7640978 | T | C | 0.1055 | -0.0392 | 0.0069 | 9.84E-09 | 32.28 | 0.00019 | TRUE | 7.22E-05 |
| rs7717505 | A | G | 0.2348 | 0.063 | 0.0044 | 1.22E-40 | 205.01 | 0.00118 | TRUE | 1.46E-29 |
| rs7774197 | C | A | 0.09894 | 0.0508 | 0.0073 | 2.97E-10 | 48.43 | 0.00029 | TRUE | 0.000139469 |
| rs7832643 | T | G | 0.405 | 0.0339 | 0.0038 | 2.67E-17 | 79.59 | 0.00048 | TRUE | 4.57E-13 |
| rs8017377 | A | G | 0.4591 | 0.0303 | 0.0038 | 2.52E-15 | 63.58 | 0.00037 | TRUE | 9.64E-11 |
| rs8044476 | G | A | 0.1293 | 0.0309 | 0.0053 | 3.53E-08 | 33.99 | 0.00020 | TRUE | 0.000179193 |
| rs8070463 | C | T | 0.5 | 0.0254 | 0.0037 | 4.28E-11 | 47.13 | 0.00027 | TRUE | 3.38E-07 |
| rs9391858 | G | A | 0.1939 | 0.0466 | 0.0052 | 8.30E-18 | 80.31 | 0.00049 | TRUE | 6.95E-11 |
| rs9875338 | A | G | 0.3879 | -0.027 | 0.0037 | 2.21E-11 | 53.25 | 0.00031 | TRUE | 3.59E-09 |
| rs9987289 | G | A | 0.9248 | 0.0714 | 0.0066 | 8.53E-24 | 117.03 | 0.00073 | TRUE | 4.02E-16 |
| Triglycerides (nsnp=68) | | | | | | | | | | |
| rs10085966 | T | C | 0.4591 | -0.03 | 0.0037 | 1.15E-13 | 65.74 | 0.00046 | TRUE | 1.75E-11 |
| rs10174692 | A | G | 0.2348 | 0.0248 | 0.0041 | 3.61E-09 | 36.59 | 0.00021 | TRUE | 1.83E-05 |
| rs10401969 | C | T | 0.07124 | -0.121 | 0.0065 | 9.70E-70 | 346.53 | 0.00196 | TRUE | 2.28E-40 |
| rs10440120 | A | C | 0.1675 | -0.0306 | 0.0044 | 5.34E-11 | 48.37 | 0.00028 | TRUE | 2.32E-07 |
| rs1062219 | T | C | 0.4921 | 0.0223 | 0.0034 | 1.69E-09 | 43.02 | 0.00024 | TRUE | 2.23E-06 |
| rs11057408 | T | G | 0.3628 | -0.0258 | 0.0035 | 2.05E-12 | 54.34 | 0.00031 | TRUE | 8.14E-09 |
| rs11613352 | T | C | 0.1913 | -0.028 | 0.0039 | 9.40E-14 | 51.55 | 0.00029 | TRUE | 0.000170679 |
| rs11752643 | T | C | 0.02639 | 0.0802 | 0.0088 | 3.96E-19 | 83.06 | 0.00053 | TRUE | 2.45E-12 |
| rs11974409 | G | A | 0.1939 | -0.0899 | 0.0042 | 1.36E-100 | 458.16 | 0.00257 | TRUE | 8.18E-67 |
| rs12123703 | G | A | 0.05673 | -0.0643 | 0.0095 | 3.54E-09 | 45.81 | 0.00053 | TRUE | 1.13E-08 |
| rs12280753 | T | C | 0.06728 | 0.1931 | 0.0064 | 1.22E-179 | 910.34 | 0.00510 | TRUE | 3.89E-129 |
| rs12630999 | A | G | 0.7361 | 0.0249 | 0.0039 | 3.15E-10 | 40.76 | 0.00023 | TRUE | 0.001506682 |
| rs12678919 | G | A | 0.1214 | -0.1702 | 0.0056 | 1.82E-199 | 923.73 | 0.00517 | TRUE | 1.94E-117 |
| rs12748152 | T | C | 0.07124 | 0.0372 | 0.0059 | 1.10E-09 | 39.75 | 0.00022 | TRUE | 8.09E-07 |
| rs13030345 | T | G | 0.1966 | 0.074 | 0.0045 | 1.02E-57 | 270.42 | 0.00152 | TRUE | 2.81E-37 |
| rs1321257 | A | G | 0.5937 | -0.0402 | 0.0034 | 5.99E-31 | 139.80 | 0.00079 | TRUE | 1.57E-17 |
| rs13389219 | T | C | 0.409 | -0.0271 | 0.0034 | 2.60E-15 | 63.53 | 0.00036 | TRUE | 2.78E-10 |
| rs1659685 | T | C | 0.7243 | 0.0503 | 0.0036 | 8.69E-40 | 195.22 | 0.00110 | TRUE | 7.75E-29 |
| rs16837533 | A | G | 0.1583 | 0.0242 | 0.0045 | 3.87E-08 | 28.92 | 0.00017 | TRUE | 0.000166355 |
| rs16948098 | A | G | 0.0409 | 0.08 | 0.0089 | 4.84E-17 | 80.80 | 0.00049 | TRUE | 1.36E-12 |
| rs17120280 | A | G | 0.128 | 0.0534 | 0.0068 | 2.78E-14 | 61.67 | 0.00068 | TRUE | 3.11E-11 |
| rs17216525 | T | C | 0.07388 | -0.1035 | 0.0061 | 1.01E-60 | 287.89 | 0.00163 | TRUE | 1.55E-35 |
| rs174535 | C | T | 0.3628 | 0.047 | 0.0034 | 1.73E-41 | 191.09 | 0.00107 | TRUE | 1.01E-26 |
| rs17513135 | T | C | 0.2322 | 0.022 | 0.0039 | 1.63E-08 | 31.82 | 0.00018 | TRUE | 4.86E-06 |
| rs1800775 | A | C | 0.4802 | -0.0396 | 0.0035 | 1.33E-26 | 128.01 | 0.00074 | TRUE | 1.28E-17 |
| rs1832007 | G | A | 0.1319 | -0.0327 | 0.0047 | 1.72E-12 | 48.41 | 0.00027 | TRUE | 1.21E-06 |
| rs2043085 | C | T | 0.6319 | -0.0327 | 0.0034 | 7.81E-20 | 92.50 | 0.00052 | TRUE | 1.96E-10 |
| rs2068888 | A | G | 0.4908 | -0.0241 | 0.0034 | 1.68E-11 | 50.24 | 0.00028 | TRUE | 1.75E-06 |
| rs2250802 | A | G | 0.6807 | 0.023 | 0.0037 | 1.21E-10 | 38.64 | 0.00022 | TRUE | 1.64E-06 |
| rs2412710 | A | G | 0.02243 | 0.0988 | 0.0132 | 1.66E-11 | 56.02 | 0.00036 | TRUE | 1.11E-09 |
| rs2652840 | C | T | 0.7665 | -0.0229 | 0.004 | 3.16E-08 | 32.78 | 0.00019 | TRUE | 3.75E-05 |
| rs2665357 | C | A | 0.5092 | 0.0212 | 0.0033 | 8.33E-10 | 41.27 | 0.00024 | TRUE | 0.000269514 |
| rs2869433 | T | C | 0.3074 | 0.0192 | 0.0034 | 4.59E-08 | 31.89 | 0.00018 | TRUE | 4.25E-05 |
| rs2954022 | A | C | 0.4697 | -0.078 | 0.0033 | 2.23E-113 | 558.68 | 0.00313 | TRUE | 4.18E-70 |
| rs3198697 | T | C | 0.3826 | -0.0198 | 0.0034 | 2.21E-08 | 33.91 | 0.00019 | TRUE | 0.001159757 |
| rs3760627 | C | T | 0.4683 | 0.0189 | 0.0034 | 5.29E-09 | 30.90 | 0.00018 | TRUE | 5.32E-05 |
| rs3761445 | A | G | 0.6148 | 0.0232 | 0.0034 | 8.06E-12 | 46.56 | 0.00026 | TRUE | 4.99E-08 |
| rs3810444 | A | T | 0.05541 | -0.0636 | 0.0076 | 1.57E-16 | 70.03 | 0.00042 | TRUE | 1.61E-07 |
| rs38855 | G | A | 0.4736 | -0.0187 | 0.0033 | 2.11E-08 | 32.11 | 0.00018 | TRUE | 0.000208813 |
| rs439401 | C | T | 0.6201 | 0.0659 | 0.0038 | 1.42E-66 | 300.75 | 0.00197 | TRUE | 1.06E-45 |
| rs4401177 | A | G | 0.1042 | 0.0687 | 0.0053 | 2.40E-37 | 168.02 | 0.00094 | TRUE | 2.75E-21 |
| rs442177 | T | G | 0.5528 | 0.0309 | 0.0033 | 1.32E-18 | 87.68 | 0.00049 | TRUE | 1.21E-13 |
| rs4587594 | A | G | 0.31 | -0.0694 | 0.0035 | 3.50E-82 | 393.17 | 0.00221 | TRUE | 7.94E-51 |
| rs4719841 | G | A | 0.3826 | 0.0232 | 0.0034 | 8.86E-11 | 46.56 | 0.00026 | TRUE | 7.14E-08 |
| rs4738684 | G | A | 0.6478 | -0.0205 | 0.0035 | 8.82E-09 | 34.31 | 0.00019 | TRUE | 2.74E-06 |
| rs4804311 | G | A | 0.1095 | -0.0392 | 0.006 | 1.49E-09 | 42.68 | 0.00025 | TRUE | 2.98E-06 |
| rs4808993 | A | C | 0.1834 | -0.0318 | 0.0053 | 1.39E-09 | 36.00 | 0.00029 | TRUE | 3.33E-07 |
| rs4810479 | T | C | 0.7124 | -0.0474 | 0.0038 | 2.07E-34 | 155.59 | 0.00088 | TRUE | 1.97E-20 |
| rs4921914 | T | C | 0.752 | -0.0353 | 0.004 | 4.87E-17 | 77.88 | 0.00045 | TRUE | 9.03E-12 |
| rs508487 | T | C | 0.08179 | 0.184 | 0.0077 | 9.23E-119 | 571.02 | 0.00330 | TRUE | 6.12E-83 |
| rs588136 | T | C | 0.7942 | -0.0495 | 0.0041 | 3.37E-30 | 145.76 | 0.00083 | TRUE | 1.20E-19 |
| rs6029143 | T | C | 0.05805 | -0.0388 | 0.0071 | 4.93E-08 | 29.86 | 0.00017 | TRUE | 0.000265939 |
| rs6066141 | C | T | 0.2414 | -0.0297 | 0.0053 | 2.34E-08 | 31.40 | 0.00035 | TRUE | 1.66E-06 |
| rs634869 | C | T | 0.562 | -0.0272 | 0.0033 | 1.78E-14 | 67.94 | 0.00038 | TRUE | 6.13E-11 |
| rs645040 | T | G | 0.7691 | 0.0293 | 0.004 | 1.83E-12 | 53.66 | 0.00030 | TRUE | 0.000193262 |
| rs676210 | A | G | 0.2309 | -0.0733 | 0.0039 | 3.28E-71 | 353.25 | 0.00198 | TRUE | 7.44E-50 |
| rs6831256 | G | A | 0.409 | 0.0258 | 0.0035 | 1.60E-12 | 54.34 | 0.00031 | TRUE | 1.91E-08 |
| rs6882076 | C | T | 0.6662 | 0.0286 | 0.0035 | 1.51E-15 | 66.77 | 0.00038 | TRUE | 6.47E-10 |
| rs6995541 | G | A | 0.3219 | 0.0265 | 0.0037 | 1.34E-12 | 51.30 | 0.00029 | TRUE | 2.27E-07 |
| rs7174819 | T | C | 0.05805 | 0.0527 | 0.0076 | 1.13E-10 | 48.08 | 0.00028 | TRUE | 1.09E-06 |
| rs719726 | T | C | 0.529 | 0.0199 | 0.0035 | 2.49E-08 | 32.33 | 0.00019 | TRUE | 0.000171236 |
| rs7248104 | A | G | 0.4169 | -0.0222 | 0.0034 | 5.04E-10 | 42.63 | 0.00024 | TRUE | 1.43E-06 |
| rs731839 | A | G | 0.6583 | -0.0224 | 0.0036 | 2.65E-09 | 38.72 | 0.00022 | TRUE | 0.000733251 |
| rs7350481 | C | T | 0.90237 | -0.2254 | 0.0066 | 1.00E-200 | 1166.33 | 0.00652 | TRUE | 6.82E-162 |
| rs749671 | A | G | 0.3945 | -0.0211 | 0.0034 | 6.11E-10 | 38.51 | 0.00022 | TRUE | 1.12E-05 |
| rs8077889 | C | A | 0.2441 | 0.0252 | 0.0042 | 9.88E-09 | 36.00 | 0.00020 | TRUE | 0.000145182 |
| rs948690 | C | T | 0.3047 | -0.0306 | 0.0052 | 6.57E-09 | 34.63 | 0.00038 | TRUE | 0.000107959 |
| rs998584 | A | C | 0.5145 | 0.0293 | 0.0037 | 3.42E-15 | 62.71 | 0.00036 | TRUE | 4.96E-08 |

HDL-C: high density lipoprotein cholesterol; LDL-C: low density lipoprotein cholesterol; TG: triglycerides; SNP: single nucleotide polymorphism; EA: effect allele; NEA: non effect allele; EAF: effect allele frequency.

Beta represents the change in lipid levels per copy of effect alle in standard deviation units. EAF were estimated from the 1000 Genomes Phase 3 panel. F statistics and R2 were estimated from beta, standard error, and sample size. Steiger test was done to check directionality of the association.

**Supplementary Table 2.** Univariable MR instrumental variables for *HMGCR*, *PCSK9*, and *NPC1L1* region extracted from Global Lipids Genetics Consortium.

| Drug target&SNP | Sample Size | EA/NEA | EAF | Effect (beta) | Standard Error | p Value | F statistic | R2 | Steiger direction | Steiger p value |
| --- | --- | --- | --- | --- | --- | --- | --- | --- | --- | --- |
| *HMGCR* |  |  |  |  |  |  |  |  |  |  |
| rs12916 | 168357 | C/T | 0.43 | -0.073 | 0.0038 | 7.80E-78 | 8023.96 | 0.0455 | TRUE | 7.68E-46 |
| rs10515198 | 173012 | A/G | 0.1 | -0.06 | 0.0061 | 6.00E-22 | 4099.80 | 0.0231 | TRUE | 5.62E-13 |
| rs12173076 | 173026 | G/T | 0.12 | -0.065 | 0.0056 | 2.00E-27 | 4627.01 | 0.0260 | TRUE | 2.35E-16 |
| rs3857388 | 172939 | C/T | 0.13 | -0.042 | 0.0059 | 2.00E-11 | 2828.26 | 0.0161 | TRUE | 1.74E-07 |
| rs7711235 | 89724 | G/A | 0.27 | -0.038 | 0.0064 | 5.00E-10 | 1902.17 | 0.0208 | TRUE | 1.11E-07 |
| *NPC1L1* |  |  |  |  |  |  |  |  |  |  |
| rs2073547 | 169889 | G/A | 0.19 | -0.049 | 0.0049 | 2.00E-21 | 4011.42 | 0.0231 | TRUE | 3.88E-12 |
| rs217386 | 173021 | G/A | 0.59 | -0.036 | 0.0038 | 1.00E-19 | 3855.81 | 0.0218 | TRUE | 4.46E-08 |
| rs7791240 | 161845 | C/T | 0.09 | -0.042 | 0.0065 | 2.00E-10 | 2605.49 | 0.0158 | TRUE | 2.66E-07 |
| *PCSK9* |  |  |  |  |  |  |  |  |  |  |
| rs11591147 | 77417 | G/T | 0.98 | -0.497 | 0.018 | 8.60E-143 | 7773.58 | 0.0913 | TRUE | 1.87E-105 |
| rs11206510 | 172812 | T/C | 0.85 | -0.083 | 0.005 | 2.38E-53 | 6634.92 | 0.0370 | TRUE | 6.44E-31 |
| rs2479409 | 172970 | G/A | 0.33 | -0.064 | 0.0041 | 2.51E-50 | 6433.03 | 0.0359 | TRUE | 3.74E-31 |
| rs585131 | 167769 | T/C | 0.82 | -0.064 | 0.005 | 2.70E-35 | 5235.17 | 0.0303 | TRUE | 2.41E-21 |
| rs11206514 | 172996 | A/C | 0.61 | -0.051 | 0.005 | 9.95E-33 | 5100.60 | 0.0286 | TRUE | 8.53E-22 |
| rs2495477 | 80151 | A/G | 0.6 | -0.064 | 0.005 | 7.28E-30 | 3346.55 | 0.0401 | TRUE | 6.29E-23 |
| rs572512 | 150564 | T/C | 0.35 | -0.048 | 0.0047 | 5.31E-26 | 4205.57 | 0.0272 | TRUE | 2.34E-17 |
| rs2479394 | 172953 | G/A | 0.29 | -0.039 | 0.0041 | 1.58E-19 | 3842.05 | 0.0217 | TRUE | 8.70E-12 |
| rs12067569 | 164264 | A/G | 0.03 | -0.089 | 0.01 | 1.97E-17 | 3516.61 | 0.0210 | TRUE | 7.56E-11 |
| rs10493176 | 86056 | T/G | 0.89 | -0.078 | 0.0102 | 2.54E-14 | 2294.57 | 0.0260 | TRUE | 2.29E-11 |
| rs11583974 | 99955 | A/G | 0.03 | -0.065 | 0.0117 | 3.95E-09 | 1896.09 | 0.0186 | TRUE | 1.45E-05 |

SNP: single nucleotide polymorphism; EA: effect allele; NEA: non effect allele; EAF: effect allele frequency.

Beta represents the change in lipid levels per copy of effect alle in standard deviation units. EAF were estimated from the 1000 Genomes Phase 3 panel. F statistics and R2 were estimated from beta, standard error, and sample size. Steiger test was done to check directionality of the association.

Supplementary Table 3. Cochran Q test of homogeneity in univariable and multivariable MR.

| Exposure | Methods | Q statistic | Degree of freedom | p Value |
| --- | --- | --- | --- | --- |
| Univariable MR |  |  |  |  |
| HDL Cholesterol | MR-Egger | 211.40 | 104 | 2.58E-09 |
|  | Inverse variance weighted | 233.67 | 105 | 8.69E-12 |
| LDL Cholesterol | MR-Egger | 185.42 | 105 | 2.17E-06 |
|  | Inverse variance weighted | 186.52 | 106 | 2.28E-06 |
| Triglycerides | MR-Egger | 117.47 | 66 | 1.00E-04 |
|  | Inverse variance weighted | 129.12 | 67 | 7.94E-06 |
| Multivariable MR |  |  |  |  |
| Pooled SNPs | MR-Egger | 484.46 | 255 | 1.98E-16 |
|  | Inverse variance weighted | 459.64 | 254 | 4.91E-14 |

HDL: high density lipoprotein; LDL: low density lipoprotein; SNP: single nucleotide polymorphism; MR-Egger: Mendelian randomization Egger method. Degree of freedom reflects the number of SNPs used in MR analysis.

Supplementary Table 4. Conditional F statistics of IVs in multivariable MR analysis.

| Conditional F statistics | nsnp | HDL-C | LDL-C | Triglycerides |
| --- | --- | --- | --- | --- |
| MR-Egger/MR IVW | 258 | 38.5283 | 57.6469 | 32.3682 |
| MR-Lasso | 228 | 34.6563 | 61.1058 | 30.2927 |

HDL-C: high density lipoprotein cholesterol; LDL-C: low density lipoprotein cholesterol; SNP: single nucleotide polymorphism; MR-Egger: Mendelian randomization Egger method; MR-Lasso: Mendelian randomization Lasso method; IVW: inverse variance weighted; nsnp: number of SNP used in model.

Supplementary Table 5. Test of heterogeneity and horizontal pleiotropy for instrumental variables in drug-target MR.

|  | Cochran Q test | | |  | MR-Egger | | |
| --- | --- | --- | --- | --- | --- | --- | --- |
| Exposure | Methods | Q statistic | p Value |  | Egger intercept | Standard Error | p Value |
| *HMGCR* | MR-Egger | 0.2976 | 0.9605 |  | 8.60E-04 | 6.10E-04 | 0.248 |
|  | IVW | 2.3458 | 0.6724 |  |  |  |  |
| *NPC1L1* | MR-Egger | 1.3852 | 0.2392 |  | -2.09E-03 | 1.70E-03 | 0.435 |
|  | IVW | 3.4757 | 0.1759 |  |  |  |  |
| *PCSK9* | MR-Egger | 8.4631 | 0.4882 |  | 2.30E-04 | 1.75E-04 | 0.229 |
|  | IVW | 10.1251 | 0.4296 |  |  |  |  |

*HMGCR*: 3-hydroxy-3-methyglutaryl-coenzyme A receptor; *NPC1L1*: Niemann-Pick C1-Like 1; *PCSK9*: proprotein convertase subtilisin/kexin type 9; IVW: inverse variance weighted; MR-Egger: Mendelian randomization Egger method.

Cochran Q test showed that there was little evidence for heterogeneity. The MR-Egger results showed the intercepts were not statistically different from 0, indicating no obvious pleiotropic effect in drug-target SNPs.

Supplementary Table 6. Causal effect estimates using SNPs of combined lipid lowering drugs on AMI in univariable MR analysis.

| Exposure | Methods | nsnp | OR (95% CI) | p Value |
| --- | --- | --- | --- | --- |
| HMGCR and NPC1L1 | Simple median | 8 | 0.993 (0.987,1.000) | 0.034 |
|  | Weighted median | 8 | 0.993 (0.988,0.999) | 0.026 |
|  | MR Egger | 8 | 0.996 (0.977,1.016) | 0.734 |
|  | IVW | 8 | 0.993 (0.988,0.998) | 0.005 |
| NPC1L1 and PCSK9 | Simple median | 14 | 0.996 (0.993,1.000) | 0.057 |
|  | Weighted median | 14 | 0.995 (0.993,0.999) | 0.006 |
|  | MR Egger | 14 | 0.996 (0.991,1.001) | 0.131 |
|  | IVW | 14 | 0.996 (0.993,0.999) | 0.004 |
| PCSK9 and HMGCR | Simple median | 16 | 0.996 (0.993,1.000) | 0.054 |
|  | Weighted median | 16 | 0.995 (0.992,0.998) | 0.002 |
|  | MR Egger | 16 | 0.994 (0.990,0.998) | 0.011 |
|  | IVW | 16 | 0.996 (0.994,0.999) | 0.001 |

*HMGCR*: 3-hydroxy-3-methyglutaryl-coenzyme A receptor; *NPC1L1*: Niemann-Pick C1-Like 1; *PCSK9*: proprotein convertase subtilisin/kexin type 9; IVW: inverse variance weighted; MR-Egger: Mendelian randomization Egger method; nsnp: number of SNPs used in analysis; OR: odds ratio; CI: confidence interval.

OR represents the effect on AMI per SD change in LDL-C predicted with genetic variants at drug target gene regions.

Supplementary Table 7. Causal effect estimates of lipid lowering drugs on AMI with univariable MR analysis.

| Exposure | Methods | nsnp | OR (95% CI) | p Value |
| --- | --- | --- | --- | --- |
| LDL-C related SNPs w/o drug-target SNPs | Simple median | 104 | 1.004 (1.002,1.007) | 6.65E-5 |
|  | Weighted median | 104 | 1.005 (1.003,1.007) | 1.32E-7 |
|  | MR-Egger | 104 | 1.006 (1.004,1.009) | 3.64E-7 |
|  | Inverse variance weighted | 104 | 1.006 (1.004,1.007) | 1.76E-13 |

*HMGCR*: 3-hydroxy-3-methyglutaryl-coenzyme A receptor; *NPC1L1*: Niemann-Pick C1-Like 1; *PCSK9*: proprotein convertase subtilisin/kexin type 9; SNP: single nucleotide polymorphism; LDL-C: low density lipoprotein cholesterol; MR-Egger: Mendelian randomization Egger method; nsnp: number of SNPs used in analysis; OR: odds ratio; CI: confidence interval.

OR represents the effect on AMI per SD change in LDL-C predicted with genetic variants excluding those related to drug-target gene regions.
